# A novel circRNA, hsa_circ_0069382, regulates gastric cancer progression

**DOI:** 10.1186/s12935-023-02871-4

**Published:** 2023-02-25

**Authors:** Haoying Wang, Hao Yuan, Qinghong Guo, Xi Zeng, Mengxiao Liu, Rui Ji, Zhaofeng Chen, Quanlin Guan, Ya Zheng, Yuping Wang, Yongning Zhou

**Affiliations:** 1 The First Clinical Medical College, Lanzhou University, Lanzhou, 730000 China; 2 Department of Gastroenterology, The First Hospital of Lanzhou University, Lanzhou, 730000 China; 3 Key Laboratory for Gastrointestinal Diseases of Gansu Province, The First Hospital of Lanzhou University, Lanzhou, 730000 China; 4 Department of Oncology Surgery, The First Hospital of Lanzhou University, Lanzhou, 730000 China

**Keywords:** circRNA, hsa_circ_0069382, miR-15a-5p, Gastric cancer, Biomarkers

## Abstract

Aberrant expression of circRNAs is closely associated with the progression of gastric cancer; however, the specific mechanisms involved remain unclear. Our aim was to identify new gastric cancer biomarkers and explore the molecular mechanisms of gastric cancer progression. Therefore, we analyzed miRNA and circRNA microarrays of paired early-stage gastric cancer samples. Our study identified a new circRNA called hsa_circ_0069382, that had not been reported before and was expressed at low levels in gastric cancer tissues. Our study also included bioinformatics analyses which determined that the high expression of hsa_circ_0069382 regulated the BTG anti-proliferation factor 2 (BTG2)/ focal adhesion kinase (FAK) axis in gastric cancer lines by sponging for miR-15a-5p. Therefore, proliferation, invasion, and migration of gastric cancer is impacted. miR-15a-5p overexpression partially restored the effects of hsa_circ_0069382. This study provides potential new therapeutic options and a future direction to explore for gastric cancer treatment, and biomarkers.

**Graphical Abstract:**

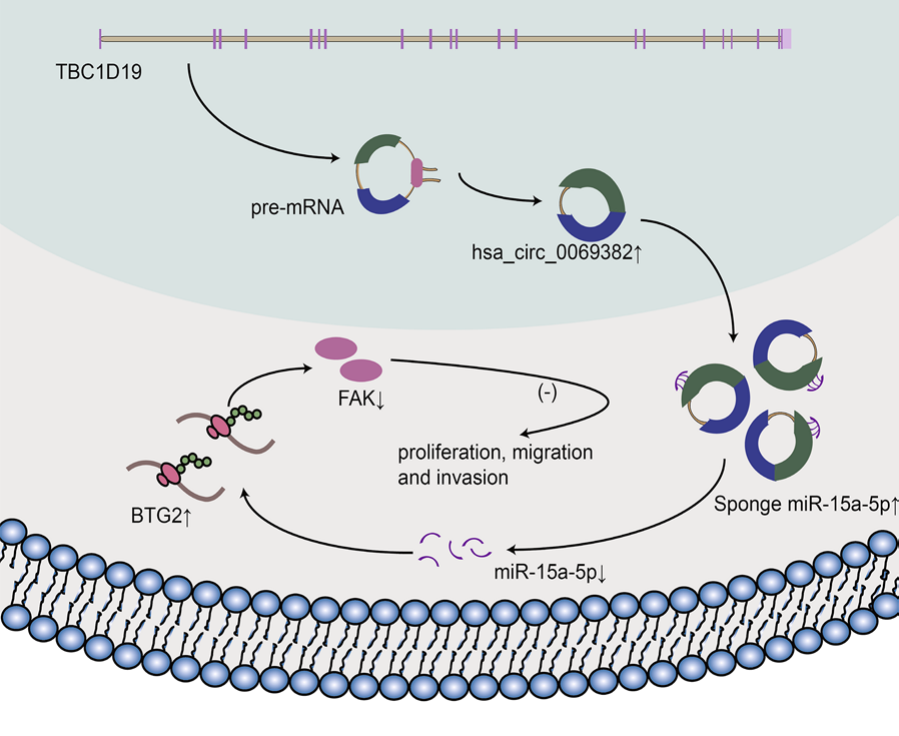

**Supplementary Information:**

The online version contains supplementary material available at 10.1186/s12935-023-02871-4.

## Introduction

According to the 2020 global cancer statistics report, among 36 cancers, gastric cancer was ranked fifth and fourth, based on its incidence rate and mortality rate, respectively [1]. The reported incidence rate was higher in males than in females [1]. In recent years, although the combination of surgery, immuno-therapy, and neoadjuvant chemotherapy has improved the survival time of patients with gastric cancer, the overall prognosis for this disease still remains poor [2, 3]. Invasion and metastasis are the main causes of mortality; however, the specific mechanisms behind these causes remain unclear. Therefore, there is an urgent need to identify effective gastric cancer biomarkers and therapeutic targets.

Non-coding genes, that constitute 98% of the human genome, consists of non-coding genes, which carry out biological functions namely, synthesizing regulatory RNAs such as, tRNA, rRNA, asRNA, snoRNA, snRNA, miRNA, and piRNA [4]. In recent years, an increasing number of studies have demonstrated that non-coding RNAs are closely associated with the occurrence and progression of gastrointestinal tumors [5, 6]. CircRNAs and miRNAs are two common non-coding RNAs that have been extensively studied in recent years. CircRNAs are closed circular non-coding RNA molecules without the 3'-poly A-tail and 5’-cap structures [7]. Although circRNAs were discovered in 1976 [8], they have been widely studied for less than a decade [9, 10]. Many studies have shown that most circRNAs originate from coding genes and form independent transcripts to regulate the biological behavior of cells [11, 12]. Biosynthesis of circRNAs occurs through a direct reverse splicing model, or lariat model, that is regulated by trans- and cis-regulatory elements [12–14]. These reverse-spliced molecules are divided into exon, intron, and exon–intron circRNAs [12, 15]. MiRNAs, ranging from 21 to 24 nucleotides in length, are a class of conserved non-coding RNA molecules [16]. The first miRNA was discovered in 1993 which was 13 years after the discovery of circRNA [17]. However, miRNA was well-studied as compared to circRNA possibly owing to the ease of isolating and detection of miRNA in various body fluids [18]. Their main mode of function is through complementary binding to the target gene 3′-UTR [19, 20]. The miRNA-mRNA binding inhibits mRNA translation, thus promoting mRNA splicing and degradation [20]. Both these RNA classes are associated with the progression of malignancies and other diseases [21–23]. CircRNAs, significant molecules of the non-coding RNA family and they function as miRNAs sponges [7]. circNRIP1, a widely studied mammalian circRNA, was found to regulate the expression of AKT1/mTOR, by sponging miR-149-5p and promote gastric cancer [24]. Additionally, circNRIP1 regulates the PTP4A1/ERK1/2 pathway by sponging miR-629-3p, thus facilitating the migration and invasion of cervical cancer [25]. The complex regulatory network between RNA and genes affects the progression of a variety of tumors such as non-small cell lung cancer and diffuse large B-cell lymphoma through various methods such as the epigenetic modification [26–28]. CircRNA and miRNA are widely used in the tumor biomarkers studies because they are highly conserved evolutionarily. They display temporal and spatial specificity, in addition to exhibiting stable expression in tissues, and blood.

To identify effective gastric cancer biomarkers and further explore the biological mechanisms of gastric cancer progression, we analyzed miRNA and circRNA microarrays of paired samples of early-stage gastric cancer cases. Our study identified a new circRNA called hsa_circ_0069382, that has not been reported before. Hsa_circ_0069382 is the product of reverse splicing of exons 4–12 of the parental gene TBC1D19. We validated our findings in vivo and in vitro, and observed that hsa_circ_0069382 regulated the expression of the BTG anti-proliferation factor 2 (BTG2)/focal adhesion kinase (FAK) axis by sponging miR-15a-5p. This further influenced the proliferation, invasion and migration of gastric cancer. Therefore, hsa_circ_0069382 and miR-15a-5p have the potential to serve as diagnostic biomarkers or therapeutic targets for gastric cancer.

## Materials and methods

### Human subjects and cell culture

The gastric cancer and paraneoplastic tissues used for array analysis and qRT-PCR were all surgically resected specimens from the Department of Oncology of the First Hospital of Lanzhou University without radiotherapy and chemotherapy. Among them, eight paired tissues for miRNA microarray (Arraystar microRNA, Kangcheng Biological Co.) and nine paired tissues for circRNA microarray (Arraystar Human circRNA Array, Kangcheng Biological Co.) were all early-stage gastric cancer tissues and paraneoplastic tissues, and 68 paired tissues for qRT-PCR were obtained from progressive gastric cancer patients. All samples were frozen in liquid nitrogen immediately after surgical excision and later transferred to − 80 °C cryogenic refrigerator for storage till use. For immunohistochemistry, gastric adenocarcinoma tissue chips were purchased (Shanghai Outdo Biotech, China) which were preserved at − 80 °C till use. This study was approved by the ethics committee of the First Hospital of Lanzhou University (LDYYLL2021-148).

Normal human gastric mucosal epithelial cells (GES-1) and human gastric cancer cell lines namely, MKN-28, SGC-7901, MGC-803, MKN-45, AGS, and HGC-27, were used (Shanghai Yuchi Biological, Shanghai, China). Human embryonic kidney (HEK) 293 T cells were also used (GenePharma Biological, Shanghai, China). All cell lines were cultured according to the manufacturer's instructions at 37 °C and 5% CO_2_.

### RNA extraction and qRT-PCR

TriQuick Reagent (Solarbio, R1100, China) was used for RNA extraction according to the manufacturer’s instructions. The Mir-X miRNA First-Strand Synthesis Kit (Takara, 638313, Japan) was used for the reverse transcription of miRNA. TransScript One-Step gDNA removal and cDNA synthesis superMix (TransScript, AT311, China) was used for reverse transcription of circRNA and the coding genes. The TransStart Top Green qPCR SuperMix (TransScript, AQ131, China) and Roche LightCycler 480 II (Roche, Switzerland) were used to perform qRT-PCR. U6 was used as an internal miRNA control and GAPDH was used as an internal control for circRNA, and the coding genes. Experiments were repeated three times independently. The primers are listed in Additional file 1: Table S5. qRT-PCR amplicons were analyzed using agarose gel electrophoresis. The Amersham Imager 680 was used for imaging gels.

### Cell transfection and lentivirus packaging

MiR-15a-5p inhibitor, inhibitor negative control (NC), miR-15a-5p mimics, and NC mimics were used (Shanghai GenePharma Biological, Shanghai, China); their sequences are listed in Additional file 1: Table S5. Using Lipofectamine 2000 (Invitrogen, USA), miR-15a-5p inhibitor, inhibitor NC, miR-15a-5p mimics, and NC mimic were transfected into SGC-7901, MGC-803, MKN-45, AGS, and HGC-27 according to the manufacturer's protocol. A plasmid concentration of 60 nM in each well of a 6-well plate for transfection was used. qRT-PCR was used to determine the transfection efficiency.

The hsa_circ_0069382 DNA sequence (673 bp) was synthesized and inserted into the pGCMV/MCS/Neo/ Kan (PEX-3) vector (GenePharma, Shanghai, China) using EcoRI and BamHI restriction sites. The hsa_circ_0069382 vector constructed by GenePharma Biological Co., Ltd., was verified by sequencing. Using Lipofectamine 2000 (Invitrogen, 11668-019, USA), the overexpression hsa_circ_0069382 and control PEX-3 plasmid were transfected into gastric cancer cells according to the manufacturer's protocol. A final plasmid concentration of 40 nM in each well of a 6-well plate was used for transfection. qRT-PCR was used to determine the transfection efficiency.

Auxiliary packaging plasmids (pGag/Pol, pRev, and pVSV-G) were co-transfected with LV3 or LV5 lentivirus plasmids into 293 T cells, to obtain lentivirus particles overexpressing the target genes (miR-15a-5p, hsa_circ_0069382). Henceforth, these plasmids will be referred to name as LV3-miR and LV5-circ, respectively (GenePharma, Shanghai, China). The lentiviral titer was determined to be 10^9^ TU/ml. A 24-well plate was seeded at a density of 2 × 10 SGC-7901 and AGS /well. When the cell confluency rate reached 40–60% in a well, lentivirus (1:50) and 5 μg/ml polybrene were added. Medium was changed at 24 h; the transfection results was observed and imaged under a fluorescence microscope (Olympus, Japan) at 48 and 72 h. The cell lines successfully transfected with the lentivirus were screened using puromycin.

### Cell proliferation assay

The Cell Counting Kit-8 (ZOMANBIO, 5BG03D, Beijing, China) was used for cell proliferation assay of the gastric cell lines. In a 100 μl volume, 5000 cells/well were seeded in a 96-well plate. The Cell Counting Kit-8 reagent (10 μL) was added to each well at 0, 24, 48, 72, and 96 h, followed by an incubation at 37 °C for 1 h. Varioskan Flash (Thermo Fisher Scientific, MA, USA) was used to measure the absorbance at 450 nm. The cell proliferation curve was evaluated based on absorbance at each time point.

### Wound healing assay

The cells were seeded in a 6-well plate. After transfection, at 90% confluency, they were scraped in a straight line with a pipette tip, washed twice with PBS and cultured in Opti-MEM (Gibco, 31985-070, USA) at 37 °C. Microscope images (Olympus, Japan) were documented at 0, 24, and 48 h after scraping. Image Pro Plus software was used to measure the scratch width. The following formulae were used to evaluate the healing rates:

24 h healing rate = (0 h scratch width−24 h scratch width)/0 h scratch width × 100%. 48 h healing rate = (0 h scratch width−48 h scratch width)/0 h scratch width × 100%.

### Transwell migration and invasion assays

Transwell migration and invasion experiments were performed using an 8 diameter transwell chamber (Corning, USA). For the invasion assay, 0.5 mg/ml Matrigel (Corning, USA) was placed in the upper chamber. After an incubation at 37 °C for 2 h, 10^6^ cells/ml cells per well were seeded in 100 µl of serum-free medium. Next, 500 μl FBS-containing medium was added to the lower chamber and incubated at 37 °C for 24 h. Cells were washed twice with PBS and fixed with methanol for 20 min (HGC-27 cells were fixed with methanol for 2 h). After staining with 0.1% crystal violet (Solarbio, G1062, China) for 20 min, the upper chamber was washed twice with PBS. The cells in the upper chamber were imaged using a microscope (Olympus, Japan). For the migration experiment, Matrigel was not added to the upper chamber; a protocol similar to the invasion assay was followed here as well.

### Flow cytometry for cell apoptosis and cell cycle assays

For cell apoptosis analysis, 400 μl binding buffer was added to 10^6^ cells/ml transfected gastric cancer cells. After adding 5 µl of Annexin V and 5 μl PI (BD, 556547, USA) each, samples were incubated for 15 min at 25 ℃ in the dark. The cells were analyzed via flow cytometry (Becton Dickinson, USA).

For the cell cycle assay, 10^6^ cells/ml of transfected were fixed overnight at 4 °C with pre-cooled 70% ethanol. Next, 500 μl staining buffer, 25 μl PI (20 ×) staining solution, and 10 μl RNase A (50 ×) (Biosharp, BL114A) were added. After incubation at 37 °C for 30 min, cells were analyzed via flow cytometry.

### Colony formation assay

The stably transfected gastric cancer cells were treated with 0.25% trypsin to form a single-cell suspension. Cells were cultured in a 10 cm culture dish at a density of 1000 cells/culture dish. After three weeks, cells were fixed for 20 min with formaldehyde. Crystal violet staining (0.1%) was performed for 15 min, after which the clones were imaged and counted.

### FISH and confocal laser scanning microscopy

FISH was used (GenePharma, F12101, Shanghai, China) to detect hsa_circ_0069382 and miR-15a-5p localization (GenePharma, Shanghai, China) in the cancer cell lines. Hsa_circ_0069382 was detected using a FAM-labelled probe; miR-15a-5p Cy3-labelled probe was used for miR-15a-5p (Additional file 1: Table S5). Additionally, DAPI (Solarbio, China) was used for nuclear staining. A laser confocal microscope (Olympus, FV3000, Japan) was used to document the images.

### RNase R treatment

Total RNA (1000 μg/μl, 4 μl) of GES-1, MKN-45, AGS, and HGC-27 was added to RNase R 2 μl (Geneseed Biotech, R0301, Guangzhou, China), 2 μl 10 × reaction buffer (Geneseed Biotech, R0301, Guangzhou, China), and 12 μl RNase-free water. The reaction mixture incubated first at 37 °C for 30 min and then at 70 °C for 10 min to inactivate the enzyme. RNase R was not added to the control. The treated RNA was subjected to reverse transcription and qRT-PCR.

### Luciferase reporter assay

Wild-type (BTG2 and hsa_circ_0069382 genes are not mutated) and mutant vectors (BTG2 and hsa_circ_0069382 genes are mutated) were constructed with pmirGlo, (GenePharma, Shanghai, China), respectively. A positive control vector (pmirGlo, GenePharma, Shanghai, China) of the miR-15a-5p inhibitor and a negative control vector (pmirGlo, GenePharma, Shanghai, China) of NC-FAM were constructed. GP-transfect mate (GenePharma, Shanghai, China) was used as the transfection reagent. We co-transfected the miR-15a-5p mimics and reporter gene vectors into 293 T cells. A dual-luciferase reporter gene detection system kit (Promega, USA) and Synergy HTx multifunctional microplate reader (Biotek, USA) were used to obtain the data.

### Western blotting

RIPA buffer (Solarbio, R0020, China) was used to extract cellular proteins. Pierce^™^ BCA Protein Assay Kit (Thermo Fisher Scientific, 23227, USA) was used for protein quantification. Proteins were separated by SDS-PAGE and transferred to PVDF membranes (Thermo Fisher Scientific, 88520 and 88518) that were then blocked with 5% skied milk powder at room temperature for 2 h. The membranes were incubated overnight at 4 °C with BTG2 (1:600, Proteintech, China), FAK (1:1000, CST, USA), and GAPDH (1:2500, CST, USA) antibodies (Additional file 1: Table S5). Next, incubation with HRP-conjugated IgG antibodies (Thermo Fisher Scientific, 1:20000, USA) was carried out at 37 °C for 1 h. ECL chemiluminescence kit (Beyotime, P0018FS, China) was used to detect the visible bands. Amersham Imager 680 was used for imaging and ImageJ software was to quantify the bands. The list of antibodies is shown in Additional file 1: Table S5.

### Immunochemistry

The gastric adenocarcinoma tissue chips (Shanghai Outdo Biotech Co., Ltd) contained 97 gastric adenocarcinoma and 83 paracancerous tissues. Immunochemistrical staining was performed with the BTG2 antibody (Abcam, 1:50, USA). The chips were scanned using a digital slide scanning system (3DHistech/Pannoramic Desk, Hungary). The data was analyzed using the Image Pro-plus software and the integrated optical density (IOD) was measured.

### In vivo metastasis assay

We purchased 30 SPF male BALB/c nude mice that were 8-week-old (GemPharmatech Biotechnology Co., Ltd. Jiangsu, China). Mice were maintained as per the SPF animal laboratory of good laboratory practice (GLP) at Lanzhou University. We state that our care for animals is in accordance with the guidelines of the First Hospital of Lanzhou University. SGC-7901 cells that were transfected with LV3, LV3-miR, LV5, and LV5-circ lentiviruses, were injected subcutaneously into the right armpit of mice (200 μl/mouse, cell suspension: matrix glue = 1:1, 5 × 10^6^ cells/200 μl). The LV3 and LV3-miR groups contained seven mice each and the LV5 and LV5-circ groups contained eight mice. The long (L) and short (S) tumor diameters were measured every three days. The 30 mice were sacrificed after three weeks and tumor volume, and weight (volume = S^2^ × L) were measured. This study was approved by the ethics committee of the First Hospital of Lanzhou University (LDYYLL2021-148).

### Bioinformatics analysis

The expression and clinical data of miR-15a-5p, BTG2 and FAK were obtained from The Cancer Genome Atlas (TCGA) database (https://portal.gdc.cancer.gov/). The ggplot2 package R (version 3.6.3) was used for analysis and visualization. miRDB (http://www.mirdb.org/) [29, 30], TargetMiner (https://www.isical.ac.in/~bioinfo_miu/targetminer20.htm) [31], TargetScan (http://www.targetscan.org/vert_72/) [32], and miRTarBase (http://mirtarbase.mbc.nctu.edu.tw/index.html) [33] were used to predict the target genes of miR-15a-5p. The Venny 2.1 online tool (https://bioinfogp.cnb.csic.es/tools/venny/index.html) and Venn Diagram package of R (version 3.6.3) were used to create Venn diagrams. The Kmplot online tool (https://kmplot.com/analysis) [34, 35] was used to analyze the survival rate of patients with gastric cancer. The MCODE, cytoHuBTG2a, and CyTargetLinker plug-ins of the Cytoscape software [36] (3.6.1) were used to construct a miRNA-mRNA interaction network and obtain the hub genes. Immunohistochemical staining images of the target genes were obtained from the Human Protein Atlas database (https://www.proteinatlas.org/). The CircBank database (http://www.circbank.cn/) [37] was used to predict the upstream circular RNA of MIR-15A-5P. Rnahybrid 2.2 (https://bibiserv.cebitec.uni-bielefeld.de/download/tools/rnahybrid.html) was used to predict the binding site of miR-15a-5p for hsa_circ_0006278, hsa_circ_0055954, and hsa_circ_0069382. The DB toolkit (http://dbtoolkit.cistrome.org/) and the AnimalTFDB (http://bioinfo.life.hust.edu.cn/AnimalTFDB/#!/) [38] databases were used to predict the upstream transcription factors of hsa_circ_0069382. The IRESite database (http://iresite.org/IRESite_web.php) [38] was used to predict the internal ribosomal entry site (IRESs) of hsa_circ_0069382. The ORF finder (https://www.ncbi.nlm.nih.gov/orffinder/) [39] was used to identify the open reading frames (ORF) of hsa_circ_0069382.

### Statistics

Experiments were repeated three times independently and the data is described as mean ± standard deviation. IBM SPSS Statistics 21.0 software was used for statistical analysis and GraphPad Prism 7 was used to illustrate the graphs. Paired Student’s *t*-tests were used to analyze the expression of 68 paired tissue samples, while unpaired Student's *t*-tests were used for the remaining data. Chi-square test was used to analyse classified data. Spearman’s correlation analysis was used to examine the potential correlations between miRNA and mRNA expressions. The following standard significance values were assigned for the statistical analysis: *p < 0.05, **p < 0.01, ***p < 0.001.

## Results

### MiR-15a-5p is upregulated in gastric cancer and is correlated with TNM cancer staging

**Fig. 1:**
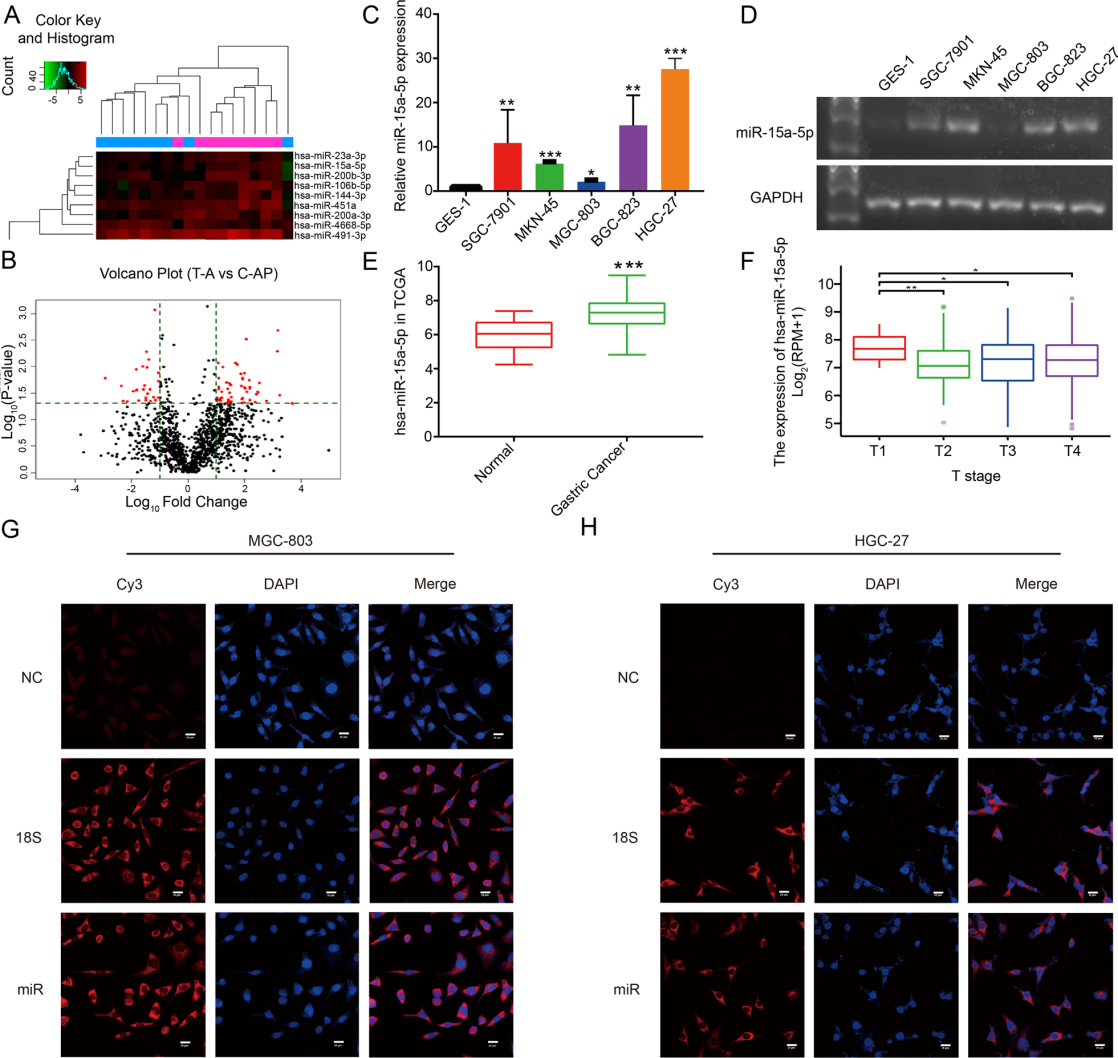
MiR-15a-5p expression in gastric cancer tissues and cells. **A** Cluster and **B **Volcano plots of the microRNA chip represent the significant differences in 81 miRNAs in gastric cancer tissues. **C** qRT-PCR and **D** agarose gel electrophoresis revealed that miR-15a-5p was expressed in GES-1 and five gastric cancer cells. TCGA data confirmed that miR-15a-5p was expressed **E** in gastric cancer tissues and **F** in T1-T4 stage gastric cancer. FISH displayed that miR-15a-5p (Cy3, red) was localized **G** in MGC-803 and **H** in HGC-27 cells. 18S (Cy3, red), was used as the positive control. Scale bar: 20 μm. *p < 0.05, **p < 0.01, and ***p < 0.001

Arraystar miRNA contained a total of 1907 human-derived miRNAs. The detection of Arraystar miRNA for 8 paired samples of early gastric cancer revealed that of 81 miRNAs, 55 were upregulated and 26 were downregulated (Fig. 1A, B). In this study, miR-15a-5p was found to be significantly upregulated in gastric cancer tissues (Fig. 1A). qRT-PCR and agarose gel electrophoresis confirmed that miR-15a-5p was highly expressed in various gastric cancer cells (Fig. 1C, D) and this finding was consistent with the TCGA database miR-15a-5p (Fig. 1E). Analysis of the TCGA data, using R programing language, revealed that miR-15-5p expression in the T1 stage was significantly higher than that in T2-T4 stages of gastric cancer (Fig. 1F). This suggests that miR-15a-5p expression levels correlated with the stages of gastric cancer. Additionally, FISH revealed a cytoplasmic localization for miR-15a-5p in the MGC-803 and HGC-27 cells (Fig. 1G, H).

### MiR-15a-5p expression regulates the proliferation, invasion, and migration of gastric tumors

We further investigated whether miR-15a-5p expression played a role in gastric cancer. We named the negative control group of miR-15a-5p inhibitor as miR-NC1, and the negative control group of miR-15a-5p mimics as miR-NC2.

**Fig. 2:**
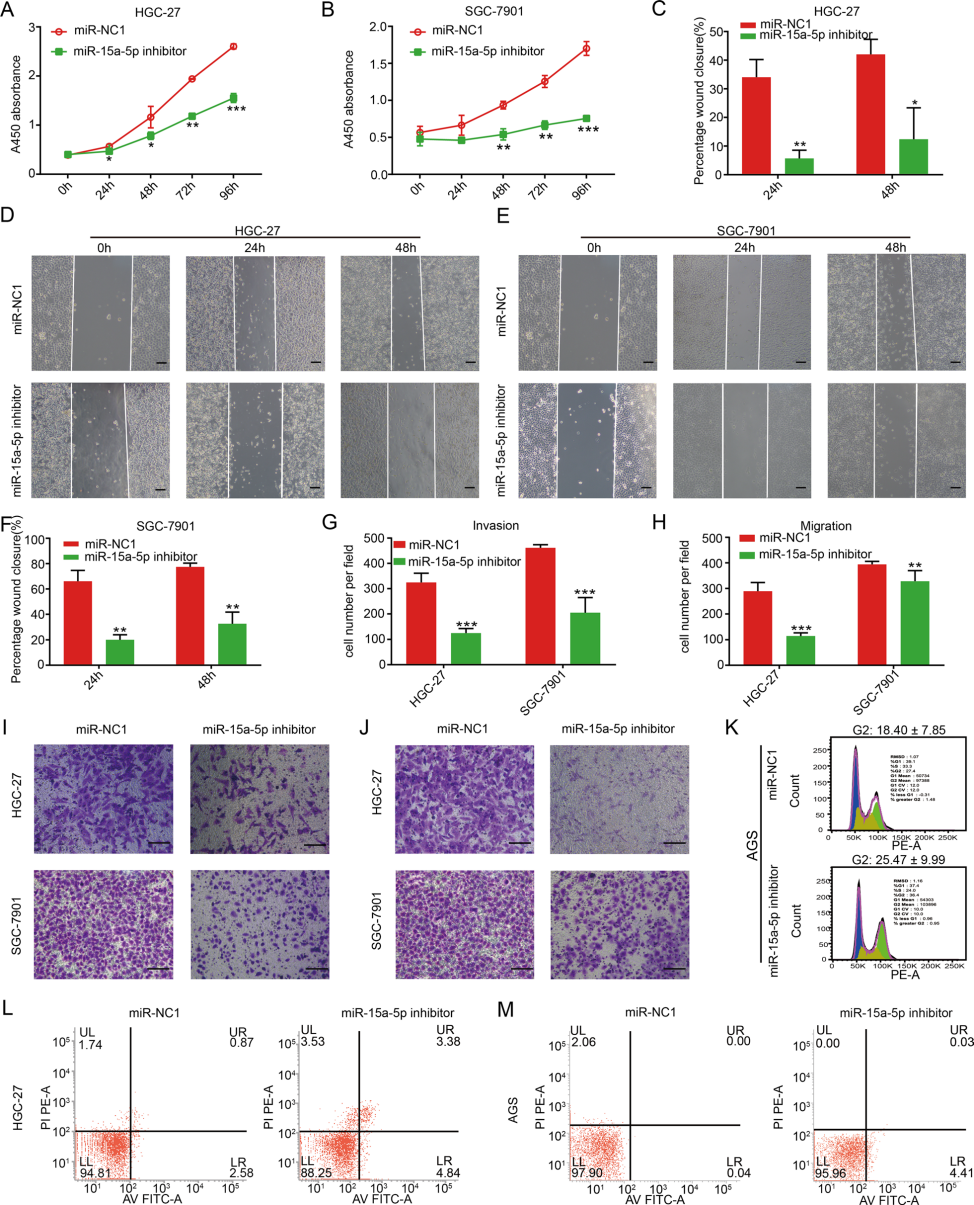
Low miR-15a-5p expression regulated the biological behavior of gastric cancer cells. CCK-8 in **A** HGC-27 and **B** SGC-7901 cells. **C** Statistical chart of D. Wound healing of **D** HGC-27 and **E** SGC-7901 cells. Scale bar, 20 μm. **F** Statistical chart of E. **G** Statistical chart of I. **H** Statistical chart of J. Transwell for the **I** invasion and **J** migration of HGC-27 and SGC-7901 cells, scale bar, 20 μm. Flow cytometry for **K** cell cycle analysis of the AGS cell line and the apoptosis of **L** HGC-27 and **M** AGS cells. *p < 0.05, **p < 0.01, and ***p < 0.001

Therefore, we used miR-15a-5p inhibitor in HGC-27, SGC-7901, and AGS cells (Additional file 1: Fig. S1A–C). As compared to the miR-NC1 group, reduction in miR-15a-5p levels significantly inhibited proliferation (Fig. 2A–B), migration (Fig. 2C–F, H, J), and invasion (Fig. 2G, I) of these cell lines. Flow cytometry analysis revealed that majority of the low miR-15a-5p AGS cells were in the G2 phase of the cell cycle, suggesting that miR-15a-5p inhibition caused a cell cycle arrest (Fig. 2K, Additional file 1: Fig. S1H). Unfortunately, the data was not statistically significantly across three experiments performed independently. (Additional file 1: Fig. S1H). Cell apoptosis assays revealed that miR-15a-5p inhibition significantly increased apoptosis of gastric cancer cells (Fig. 2L, M, Additional file 1: Fig. S1G).

**Fig. 3:**
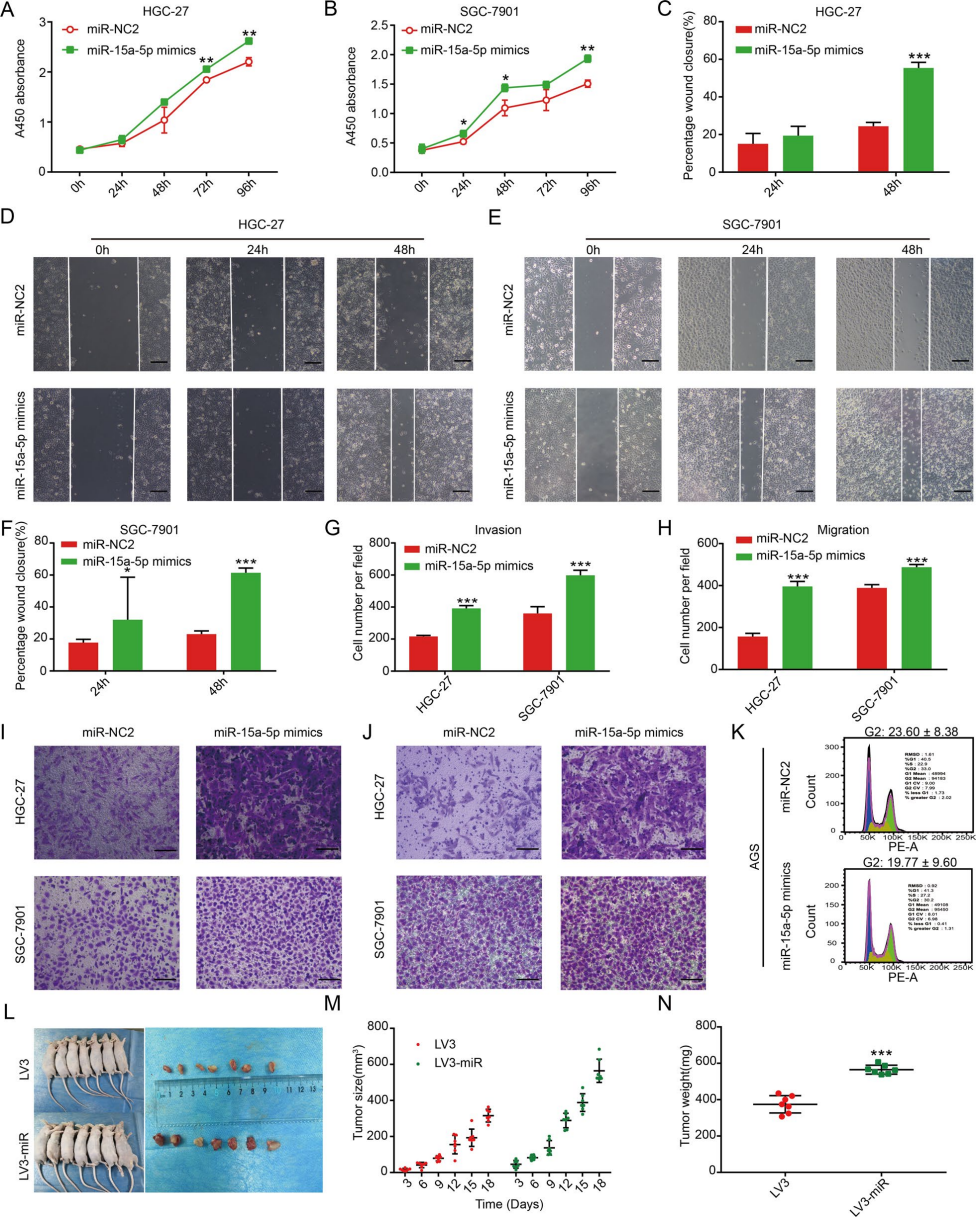
High expression of miR-15a-5p regulated the biological behavior of gastric cancer cells in vitro and in vivo. CCK-8 of **A** HGC-27 and **B** SGC-7901 cells. **C** Statistical chart of D. Wound healing of **D** HGC-27 and **E** SGC-7901cells, scale bar, 20 μm. **F** Statistical chart of E. **G** Statistical chart of I. **H** Statistical chart of J. Transwell for the **I** invasion and **J** migration of HGC-27 and SGC-7901 cells, scale bar, 20 μm. **K** Flow cytometry for the cell cycle of AGS. **L** Xenograft tumors were obtained from mice injected with SGC-7901 cells transfected with LV3 and LV3-miR (n = 7), the **M** size and **N** weight of xenograft tumors. *p < 0.05, **p < 0.01, and ***p < 0.001

Transfection of miR-15a-5p mimics in cell lines, with high miR-15a-5p expression (Additional file 1: Fig. S1D–F) and miR-15a-5p overexpression promoted cell proliferation (Fig. 3A–B), migration (Fig. 3C–F, H, J), and invasion (Fig. 3G, I). Flow cytometry demonstrated that as compared to the miR-NC2 group, the number of cells in the G2 phase, with high miR-15a-5p expression, was decreased, thus suggestive of increased proliferation (Fig. 3K, Additional file 1: Fig. S1H). Unfortunately, this data was not statistically significant (Additional file 1: Fig. S1H). The LV3 empty vector (Additional file 1: Fig. S3A) and miR-15a-5p overexpression vector (LV3-miR) were transfected into AGS cells (Additional file 1: Fig. S3C and 3H). In the high miR-15a-5p expression group, AGS cells displayed a stronger ability to form colonies (Additional file 1: Fig. S3E, F), suggesting that this group of cells has a stronger proliferative ability.

To study the effect of miR-15a-5p in vivo, we constructed SGC-7901 cell model with miR-15a-5p overexpression as a basis for the xenograft tumor model. The LV3 empty (Additional file 1: Fig. S3A) and miR-15a-5p overexpression (LV3-miR) vectors were transfected into SGC-7901 cells (Additional file 1: Fig. S3D and 3H). Figure 3L displays the tumors observed in mice belonging to both the LV3 and LV3-miR groups (n = 7). The size and weights of tumors in the LV3-miR group mice were larger as compared to the LV3 group. This indicated that miR-15a-5p promoted the growth of allogeneic tumors in vivo (Fig. 3M, N).

### Hsa_circ_0069382 interacts directly with miR-15a-5p

**Fig. 4:**
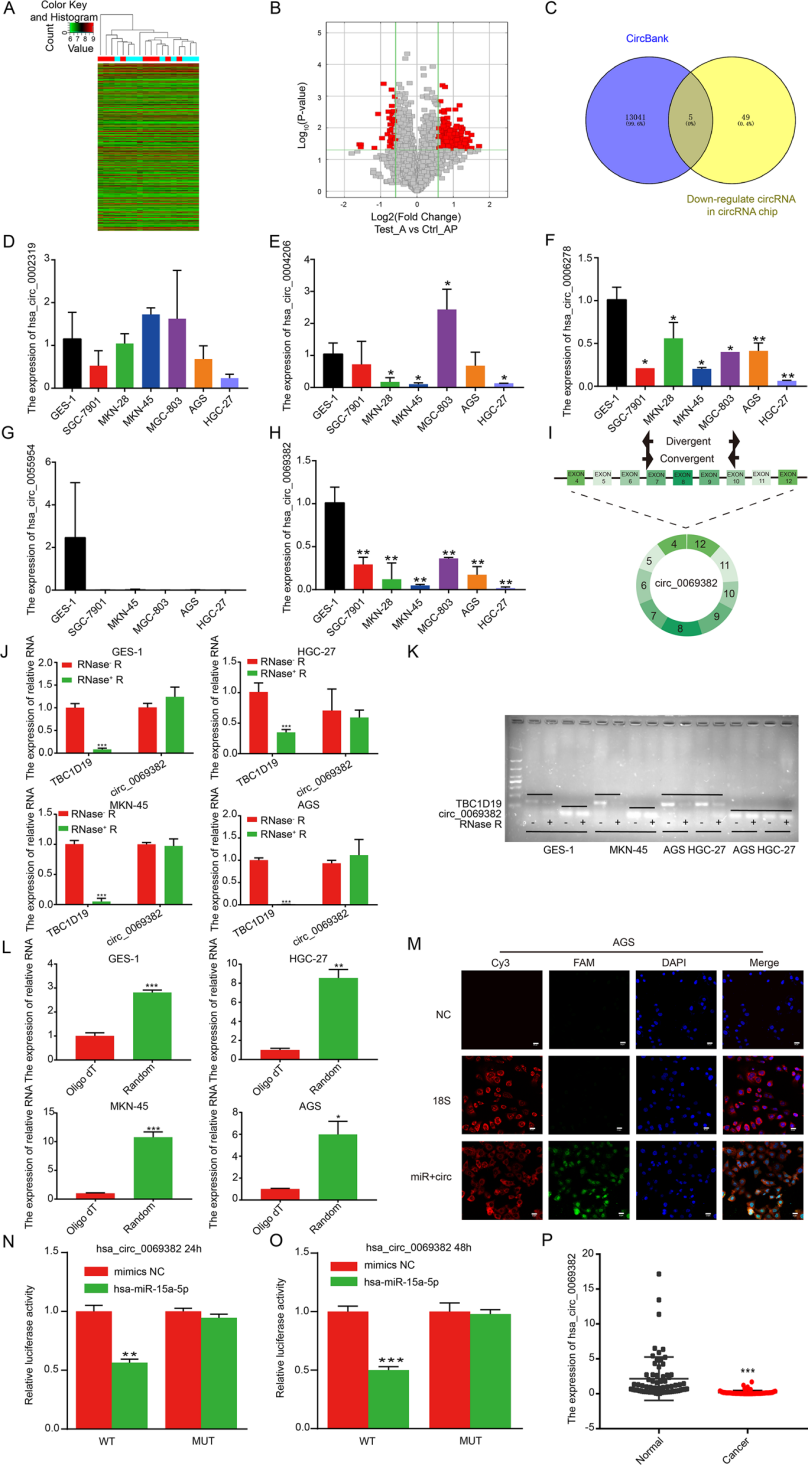
hsa_circ_0069382 is a new circRNA that interacts directly with miR-15a-5p. **A** Cluster and **B** volcano plots of the circRNA chip represent significant differences in 266 circRNAs of gastric cancer tissues. **C** Venn chart for the upstream regulatory genes of miR-15a-5p. qRT-PCR showed the expression of **D** hsa_circ_0002319, **E** hsa_circ_0004206, **F** hsa_circ_0006278, **G** hsa_circ_0055954, and **H** hsa_circ_0069382 in GES-1 cells and six gastric cancer cell lines. **I** Schematic diagram of hsa_circ_0069382 and the PCR primers sequence location. **J** qRT-PCR and **K** agarose gel electrophoresis confirmed that hsa_circ_0069382 resisted RNase R digestion. **L** qRT-PCR for hsa_circ_0069382 expression in the random primer group and in oligo dT group. **M** FISH confirmed the co-localization of hsa_circ_0069382 (FAM, green) and miR-15a-5p (Cy3, red) in AGS. Cytoplasmic 18S (Cy3, red), was used as a positive control, scale bar, 20 μm. Luciferase reporter gene assays at **N** 24 h and **O** 48 h confirmed that miR-15a-5p bound to hsa_circ_0069382. **P** qRT-PCR examined hsa_circ_0069382 expression in 68 paired para-cancer and gastric cancer tissues. *p < 0.05, **p < 0.01, and ***p < 0.001

Arraystar circRNA analysis demonstrated that of the 266 circRNAs, 212 were significantly upregulated and 54 were significantly downregulated in gastric cancer tissues (Fig. 4A, B). To identify the molecular mechanisms regulating miR-15a-5p, the circBank database was used to predict the potential upstream circular RNAs involved and compared this data with results from a previous circRNA chip analysis and identified five circRNA molecules (Fig. 4C). We also evaluated the expression of these circRNAs namely, hsa_circ_0002319, hsa_circ_0004206, hsa_circ_0006278, hsa_circ_0055954 and hsa_circ_0069382 by qRT-PCR (Fig. 4D–H). As compared to GES-1, there was no difference in the expression of hsa_circ_0002319 in gastric cancer cells (Fig. 4D). hsa_circ_0004206 was expressed at low levels in MKN-28, MKN-45, and HGC-27 cells, at high levels in MGC-803 cells; it was not altered in SGC-7901, and AGS cells (Fig. 4E). In all cell lines, hsa_circ_0006278, hsa_circ_0055954, and hsa_circ_0069382 were expressed at low levels (Fig. 4F–H). Rnahybrid 2.2 was used to predict the binding site of miR-15a-5p for hsa_circ_0006278, hsa_circ_0055954, and hsa_circ_0069382. We also evaluated the minimum free energy (MFE), binding site, and seed sequence length (Additional file 1: Table S3). Hsa_circ_0069382 was found to have the largest MFE and longest seed sequence among the three circular RNA molecules, suggesting that it was more probable that it had a direct effect on miR-15a-5p (Additional file 1: Table S3).

Figure 4I shows hsa_circ_0069382 is formed by the reverse splicing of exons 4–12 of TBC1D19. Convergent and divergent primers were used to detect TBC1D19 and hsa_circ_0069382 (Fig. 4I). By qRT-PCR to detect RNA after RNase R treatment, we found that hsa_circ_0069382 tolerated the digestion by RNase R in GES-1 and gastric cancer cells, whereas its homologous linear molecule TBC1D19 was digested (Fig. 4J). Similar results were confirmed using agarose gel electrophoresis (Fig. 4K). We used oligo dT and random primers to reverse transcribe total RNA of GES-1, MKN-45, AGS, and HGC-27 cell lines and next, we performed qRT-PCR. Among the four cell lines, hsa_circ_0069382 expression in the random primer group was higher than in the oligo dT primer group (Fig. 4L). This indicated that hsa_circ_0069382 had no poly-A tail, thus confirming that hsa_circ_0069382 is a circular RNA molecule. No difference in the homologous linear molecule TBC1D19 expression was detected among the tested cell lines (Additional file 1: Fig. S2A).

FISH results confirmed that hsa_circ_0069382 was located both in the nucleus and cytoplasm (Fig. 4M). Among FISH results, hsa_circ_0069382 was colocalized with miR-15a-5p in the cytoplasm (Fig. 4M). Luciferase reporter gene assays at 24 h and 48 h revealed that luciferase activity decreased significantly after miR-15a-5p was transfected into the hsa_circ_0069382 wild-type group, while it remained unchanged for the hsa_circ_0069382 mutant group (Fig. 4N, O, Additional file 1: Fig. S2 B, C). The complementary sequence of the binding site of hsa_circ_0069382 with miR-15a-5p is shown in Additional file 1: Table S3. These results indicated that hsa_circ_0069382 interacts with miR-15a-5p.

qRT-PCR results of 68 pairs of gastric cancer and adjacent tissues confirmed that hsa_circ_0069382 was expressed at low levels in gastric cancer tissues (Fig. 4P). Additional file 1: Table S2 shows the clinical information of the 68 gastric cancer patients. Table 1 shows the correlation between hsa_circ_0069382 expression and clinicopathological parameters of gastric cancer. The results demonstrated that the expression of hsa_circ_0069382 was not associated with the location, TNM stage, lymph node metastasis, Lauren gastric cancer classification, or differentiation (Table 1).

**Table 1:** Correlation between hsa_circ_0069382 expression and clinicopathological parameters of gastric cancer

	hsa_circ_0069382 high expression (n = 8)	hsa_circ_0069382 low expression (n = 60)	P value
Age (years)			
≤ 50	0(0.0%)	6(10.0%)	
> 50	8(100.0%)	54(90.0%)	0.349
Gender			
Male	6(75.0%)	44(73.3%)	
Female	2(25.0%)	16(26.7%)	1.000
Location			
Gastric corpus	0(0.0%)	9(15.0%)	
Gastric antrum	3(37.5%)	27(45.0%)	
Others	5(62.5%)	24(40.0%)	0.345
TNM stage			
I–II	5(62.5%)	21(35.0%)	
III-IV	3(37.5%)	39(65.0%)	0.264
Lymph node metastasis			
Yes	0(0.0%)	1(1.4%)	
No	8(100.0%)	69(98.6%)	1.000
Lauren classification			
Diffuse-type	2(25.5%)	17(28.3%)	
Intestinal-type	1(12.5%)	6(10.0%)	
Mixed	2(25.0%)	21(35.0%)	
Unknown	3(37.5%)	16(26.7%)	0.902
Degree of differentiation			
Poorly	2(25.0%)	23(38.3%)	
Moderate	0(0.0%)	5(8.3%)	
Moderate-poorly	5(62.5%)	29(48.3%)	
Unknown	1(12.5%)	3(5.0%)	0.579

### Hsa_circ_0069382 regulates the proliferation, invasion, and migration of tumor through miR-15a-5p

To investigate whether hsa_circ_0069382 regulates the proliferation, invasion, and migration of gastric cancer cells by sponging miR-15a-5p, we transfected HGC-27 and AGS cells with an hsa_circ_0069382 overexpression plasmid. An empty PEX-3 plasmid was used as a control. qRT-PCR results confirmed successful transfection. the expression of hsa_circ_0069382 within the hsa_circ_0069382 plasmid group increased significantly (Additional file 1: Fig. S2D, E, G). However, the expression of its homologous linear molecule, TBC1D19 remained unchanged (Additional file 1: Fig. S2F, H). Furthermore, qRT-PCR confirmed that the high hsa_circ_0069382 expression downregulated miR-15a-5p expression in these cells (Additional file 1: Fig. S2I, J).

**Fig. 5:**
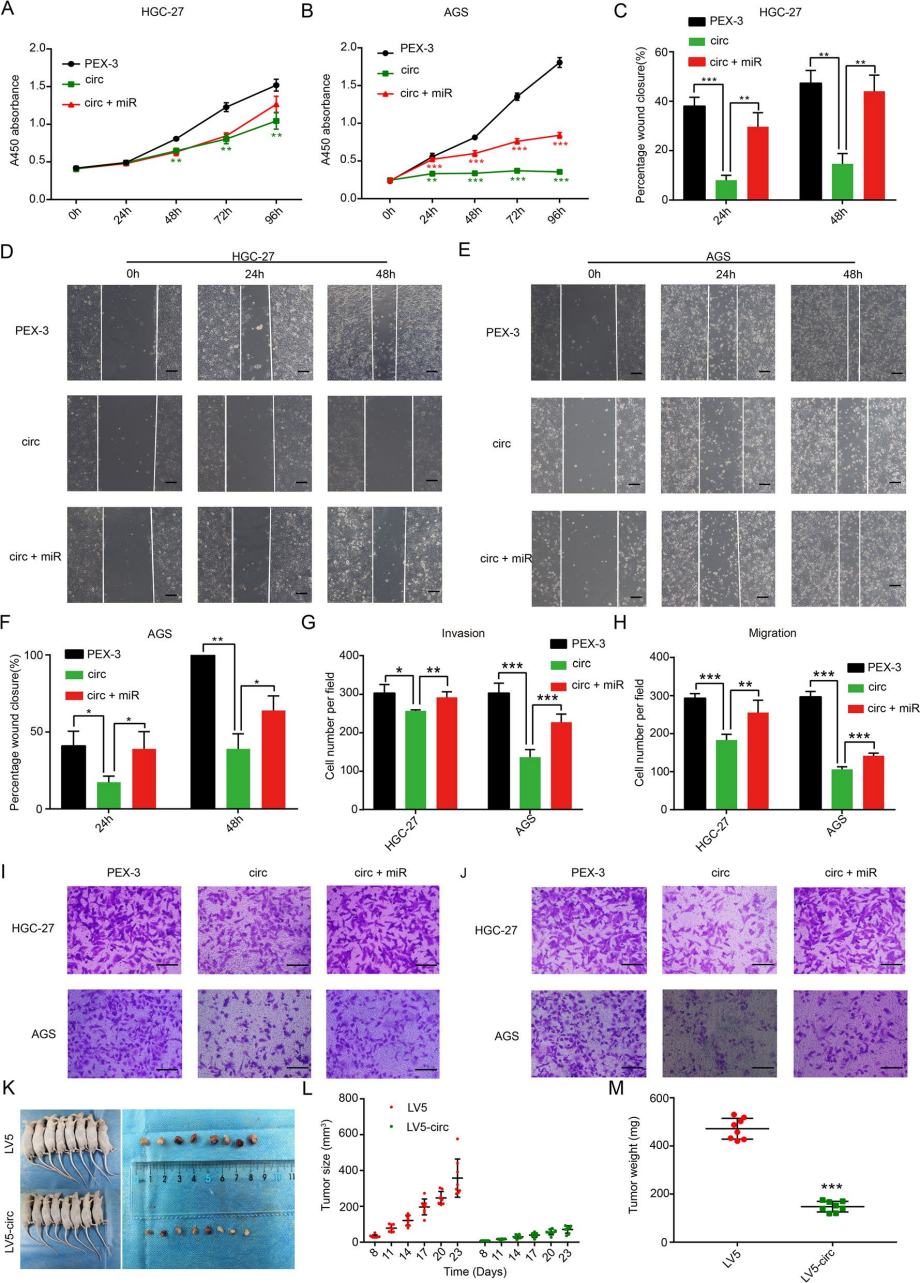
High expression of hsa_circ_0069382 regulated the biological behavior of gastric cancer cells in vivo and in vitro, and miR-15a-5p partially restored the effect of hsa_circ_0069382. CCK-8 of **A** HGC-27 and **B** AGS cells. **C** Statistical chart of D. Wound healing of **D** HGC-27 and **E** AGS cells, scale bar, 20 μm. **F** Statistical chart of E. **G** Statistical chart of I. **H** Statistical chart of J. Transwell for the **I** invasion and **J** migration of HGC-27 and AGS cells, scale bar, 20 μm. **K** Mice were injected with SGC-7901 cells transfected with LV5 and LV5-circ and xenograft tumors were obtained from the mice. **L** The size and (M) weight of xenograft tumors. *p < 0.05, **p < 0.01, and ***p < 0.001

High hsa_circ_0069382 expression inhibited the proliferation (Fig. 5A, B), migration (Fig. 5C–F, H, J), and invasion (Fig. 5G, I) of HGC-27 and AGS cells. High miR-15a-5p expression inhibited the effect of high hsa_circ_0069382 levels (Fig. 5A–J). The LV5 empty vector (Additional file 1: Fig. S3B) and the hsa_circ_0069382 overexpression vector (LV5-circ) was transfected into AGS cells (Additional file 1: Fig. S3C, I). AGS cells had reduced colony formation ability in the hsa_circ_0069382 high expression group than in transfected with PEX group (Additional file 1: Fig. S3E, G).

To study the effect of hsa_circ_0069382 in vivo, we constructed an SGC-7901 cell model with hsa_circ_0069382 overexpression and a xenograft tumor model. The lentivirus-packaged LV5 empty vector (Additional file 1: Fig. S3B) and hsa_circ_0069382 overexpression vector (LV5-circ) was transfected into SGC-7901 cells. Additional file 1: Fig. S3D and 3I show the transfection results. Figure 5K shows the mice in the LV5 and LV5-circ groups on the left (n = 8) and the tumors obtained at the sacrifice of the two groups of mice on the right. Compared with the LV5 group, the tumor size and weight of the LV5-circ group mice were smaller, indicating that hsa_circ_0069382 inhibited the growth of allogeneic tumors in vivo (Fig. 5L, M).

### BTG2/FAK is a downstream target protein of hsa_circ_0069382/miR-15a-5p

**Fig. 6:**
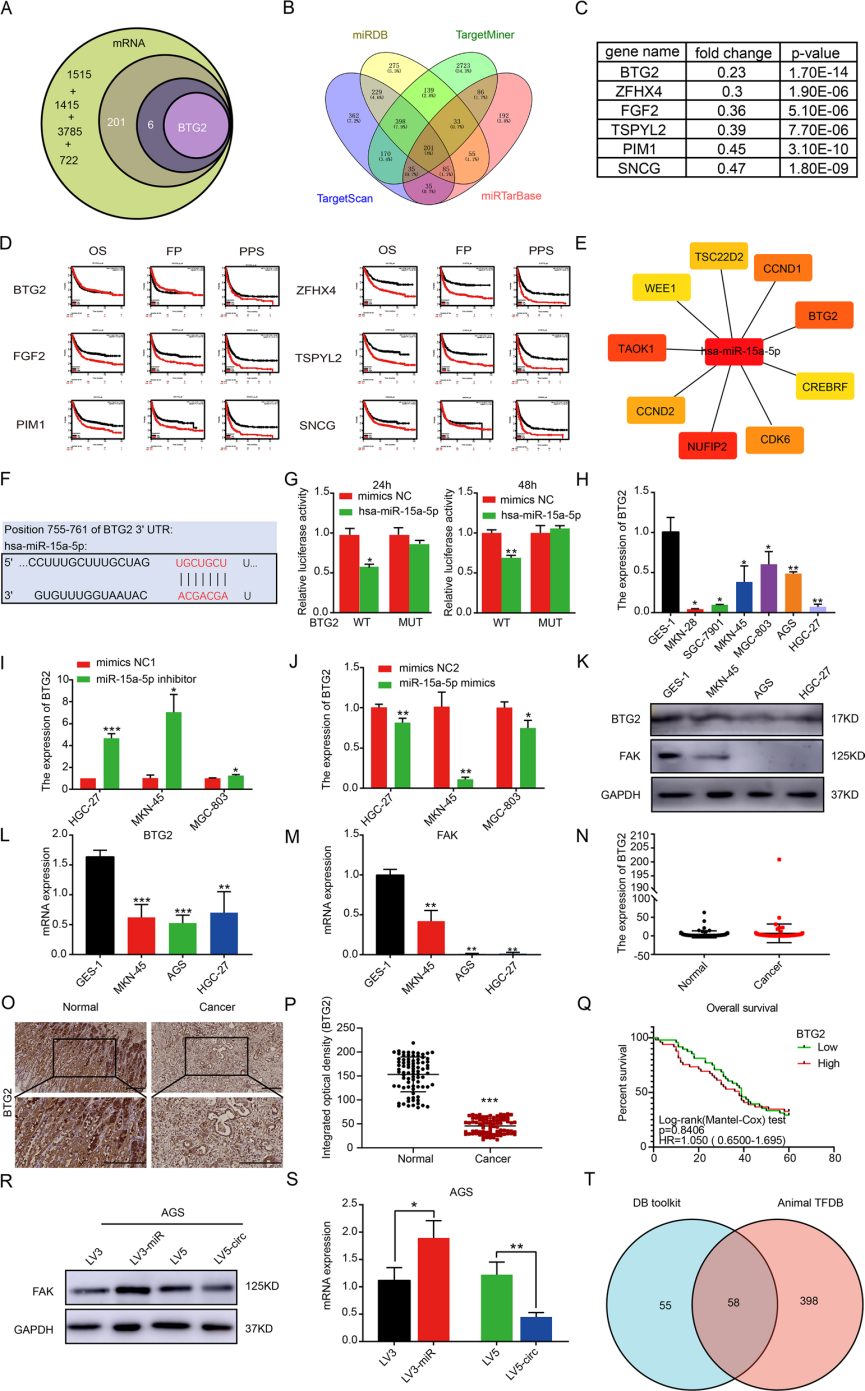
BTG2 is a downstream target molecule of miR-15a-5p. **A** The flow chart by adobe illustrator software represents the approach for identifying BTG2. **B** The Venn diagram of the miR-15a-5p target genes obtained from four databases (miRDB, TargetMiner, TargetScan, miRTarBase). **C** The fold changes of six genes among the 201 intersecting target genes < 0.5, *P* value < 0.05. **D** Survival analysis of six genes in gastric cancer, including overall survival (OS), free-progression survival (FP), and post-progression survival (PPS). **E** Nine hub genes were obtained by Cytoscape software. **F** The binding site diagram of miR-15a-5p to BTG2. **G** Luciferase reporter gene assays at 24 h and 48 h confirmed that miR-15a-5p bound to the 755–761 site of BTG2. qRT-PCR showed that BTG2 was expressed **H** in six gastric cancer cell lines, **I** in the low expression of miR-15a-5p and **J** high expression of miR-15a-5p in HGC-27, MKN-45, and MGC-803 cells. **K** Western Blot for BTG2 and FAK expression in GES-1, MKN-45, AGS, and HGC-27 cells. **L** and **M** Statistical chart of K. **N** qRT-PCR of BTG2 expression in 68 paired para-cancer and gastric cancer tissues. **O** Immunohistochemical of BTG2 expression in gastric cancer tissue chips, scale bar, 20 μm. **P** Statistical chart of O. **Q** OS of BTG2 in gastric cancer tissue chips. **R** Western Blot for FAK levels in the AGS cells with miR-15a-5p and hsa_circ_0069382 overexpression **S** Statistical chart of R. **T** Venn chart of transcription factors for hsa_circ_0069382 from the DB toolkit and Animal TFDB database. p < 0.05, **p < 0.01, and ***p < 0.001

To identify the putative downstream molecules of hsa_circ_0069382/miR-15a-5p, we performed bioinformatics analysis. Figure 6A shows the identification of the target gene BTG2 of miR-15a-5p. The miRDB, TargetMiner, TargetScan, and miRTarBase databases were used to predict the target genes of miR-15a-5p. We identified 1415, 3785, 1515, and 722 target genes, from these four databases, respectively (Fig. 6B). All 7437 target genes obtained were compared and 201 common genes were obtained (Fig. 6B, Additional file 1: Table S1). Among these, the fold changes of BTG2, zinc finger homeobox 4 (ZFHX4), fibroblast growth factor 2 (FGF2), TSPY Like 2 (TSPYL2), Pim-1 proto-oncogene (PIM1), and synuclein gamma (SNCG) were less than 0.5 (p < 0.05) (Fig. 6C). The Kmplot software was used to estimate the survival analysis in gastric cancer based on the expression of these six genes. We evaluated the overall survival (OS), free-progression survival (FPS), and post-progression survival (PPS). Only the high expression of BTG2 had better FPS, while the increased expression of the other five genes was all related to poor survival (Fig. 6D). The MCODE, cytoHuBTG2a, and CyTargetLinker plug-ins of the Cytoscape software were used to construct an interaction network for miR-15a-5p and the 201 target genes. We obtained nine hub genes of miR-15a-5p (Fig. 6E), of which BTG2 interacted with miR-15a-5p (Fig. 6E). Figure 6F shows the binding positions of miR-15a-5p and BTG2 3'-UTRs. The luciferase reporter gene assays showed that the luciferase activity of the BTG2 wild-type group was significantly reduced after miR-15a-5p mimic transfection at 24 and 48 h. Additionally, no change was detected for the BTG2 755–761 site mutation group after miR-15a-5p mimic transfection (Fig. 6G, Additional file 1: Fig. S1I, J). These findings confirmed that miR-15a-5p directly binds to BTG2.

qRT-PCR results showed that as compared to GES-1, BTG2 expression was significantly lower in six gastric cancer cell lines (Fig. 6H). This was consistent with BTG2 expression levels in paired and unpaired gastric cancer samples in TCGA (Additional file 1: Fig. S1K, L). The low expression of miR-15a-5p resulted in upregulation of BTG2 in the cell lines, whereas its high expression inhibited BTG2 (Fig. 6I, J). Western blot analysis confirmed low expression levels of BTG2 in gastric cancer cells (Fig. 6K, L). Focal adhesion kinase FAK (also named PTK2, protein tyrosine kinase 2) is regulated by BTG2 [40, 41]. We evaluated FAK by western blotting and confirmed its low expression in MKN-45, AGS and HGC-27 (Fig. 6K, M). Correlation analysis of miRNA and mRNA in TCGA database showed that miR-15a-5p negatively correlated with BTG2 expression, and miR-15a-5p positively correlated with FAK expression in gastric cancer (Additional file 1: Fig. S1M, N). Immunohistochemical staining results obtained from the Human Protein Atlas database demonstrated that FAK is highly expressed in gastric cancer (F Additional file 1: Fig. S1O).

qRT-PCR results of 68 pairs of gastric cancer and adjacent tissues showed that BTG2 expression was not significantly different between them (Fig. 6N), possibly because of the small number of tissue samples. Additional file 1: Table S2 shows the clinical features of the 68 gastric cancer patients. To expand the sample size, we purchased gastric cancer tissue chips for immunohistochemical staining. This microarray contains 180 samples, of which, 83 pairs are paired samples of gastric cancer and para-cancerous tissue, and 14 separate samples of gastric adenocarcinoma. We observed that in 83 pairs of samples, the BTG2 expression in cancer tissue was significantly lower than that in adjacent tissues (Fig. 6O, P). Survival analysis for BTG2 expression was performed in 97 cancer samples using the median method; the expression BTG2 did not impact the survival time of patients (Fig. 6Q). This may be due to the fact that all 97 gastric cancer samples from tissue microarrays used for the survival analysis were progressive gastric adenocarcinoma samples. Table 2 presents the baseline characteristics of the 97 samples used in the tissue microarray. Univariate and multivariate COX regression analyses were performed on the clinical data of these 97 samples. It was found that age, T, N, M stage, tumor diameter, and AFP were risk factors that influenced the post-operative survival time of the patients (Table 3).

**Table 2:** The baseline characteristics of 97 gastric cancer samples used in the tissue microarray

Characteristic	Low expression of BTG2	High expression of BTG2	p
n	48	49	
Gender, n (%)			0.674
Female	10 (10.3%)	13 (13.4%)	
Male	38 (39.2%)	36 (37.1%)	
T stage, n (%)			0.969
T1	5 (5.2%)	4 (4.1%)	
T2	8 (8.2%)	9 (9.3%)	
T3	22 (22.7%)	24 (24.7%)	
T4	13 (13.4%)	12 (12.4%)	
N stage, n (%)			0.363
N0	11 (11.3%)	16 (16.5%)	
N1	6 (6.2%)	8 (8.2%)	
N2	16 (16.5%)	9 (9.3%)	
N3	15 (15.5%)	16 (16.5%)	
M stage, n (%)			0.249
M0	44 (45.4%)	40 (41.2%)	
M1	4 (4.1%)	9 (9.3%)	
Pathologic stage, n (%)			0.085
II	15 (15.6%)	8 (8.3%)	
II–III	15 (15.6%)	13 (13.5%)	
III	17 (17.7%)	28 (29.2%)	
Degree of differentiation, n (%)			0.084
Moderate	23 (23.7%)	15 (15.5%)	
Poorly	20 (20.6%)	29 (29.9%)	
Unknown	1 (1%)	4 (4.1%)	
Well	4 (4.1%)	1 (1%)	
Tumor diameter(cm), n (%)			0.783
> 5	7 (7.4%)	8 (8.4%)	
≤ 3	17 (17.9%)	21 (22.1%)	
3–5	22 (23.2%)	20 (21.1%)	
Location, n (%)			0.099
Cardia	0 (0%)	2 (2.1%)	
Cardia-corpus	1 (1%)	1 (1%)	
Cardia-fundus	9 (9.3%)	3 (3.1%)	
Fundus	0 (0%)	1 (1%)	
Gastric antrum	29 (29.9%)	36 (37.1%)	
Gastric corpus	9 (9.3%)	4 (4.1%)	
Others	0 (0%)	2 (2.1%)	
Surgical methods, n (%)			0.561
Distal gastrectomy	31 (32.3%)	33 (34.4%)	
Proximal gastrectomy	9 (9.4%)	7 (7.3%)	
total gastrectomy	8 (8.3%)	6 (6.2%)	
others	0 (0%)	2 (2.1%)	
H pylori infection, n (%)			1.000
(−)	20 (27%)	17 (23%)	
( +)	21 (28.4%)	16 (21.6%)	
Age, mean ± SD	59.79 ± 10.05	58.31 ± 10	0.467
AFP, meidan (IQR)	2.4 (1.23, 3.88)	2.59 (1.5, 3.79)	0.799
CEA, meidan (IQR)	2.09 (1.15, 3.37)	2.16 (1.42, 3.13)	0.579
CA199, meidan (IQR)	13.65 (8.06, 29.7)	10.84 (6.94, 24.02)	0.308
CA153, meidan (IQR)	9.1 (6.34, 14.65)	5.9 (4.32, 13.83)	**0.041**
CA125, meidan (IQR)	6.7 (4.55, 12.05)	7.8 (3.95, 12.3)	0.951
CA50, meidan (IQR)	5.96 (2.88, 12.75)	2.66 (1, 9.86)	0.084

**Table 3:** Univariate and multivariate COX regression analysis of 97 gastric cancer samples used in the tissue microarrays

Characteristics	Tota l(N)	Univariate analysis		Multivariate analysis	
		Hazard ratio (95% CI)	P value	Hazard ratio (95% CI)	P value
Age	97	1.030 (1.004–1.057)	**0.022**	1.052 (1.015–1.090)	**0.006**
Gender	97				
Male	74	Reference			
Female	23	1.070 (0.617–1.857)	0.809		
T stage	97				
T1	9	Reference			
T2	17	1.441 (0.373–5.574)	0.596	0.883 (0.172–4.536)	0.881
T3	46	3.515 (1.076–11.486)	**0.037**	1.234 (0.280–5.446)	0.781
T4	25	9.291 (2.754–31.351)	**< 0.001**	2.270 (0.471–10.936)	0.307
N stage	97				
N0	27	Reference			
N1	14	0.680 (0.266–1.737)	0.420	0.751 (0.239–2.356)	0.624
N2	25	1.547 (0.788–3.040)	0.205	1.348 (0.605–3.005)	0.465
N3	31	2.793 (1.492–5.228)	**0.001**	2.058 (0.816–5.192)	0.126
M stage	97				
M0	84	Reference			
M1	13	8.343 (4.140–16.812)	**< 0.001**	5.224 (1.856–14.704)	**0.002**
Pathologic stage	96				
II	23	Reference			
II–III	28	1.293 (0.678–2.463)	0.435		
III	45	1.029 (0.559–1.895)	0.927		
Differentiation	97				
Poorly	49	Reference			
Moderate	38	0.683 (0.404–1.155)	0.155		
Well	5	0.466 (0.143–1.517)	0.205		
Unknown	5	1.501 (0.586–3.847)	0.398		
Tumor diameter(cm)	95				
≤ 3	38	Reference			
3–5	42	1.366 (0.799–2.333)	0.254	1.085 (0.577–2.037)	0.801
> 5	15	2.259 (1.127–4.531)	**0.022**	1.108 (0.414–2.962)	0.839
Location	97				
Cardia	2	Reference			
Cardia-corpus	2	79237078.175 (0.000-Inf)	0.996		
Cardia-fundus	12	25653907.317 (0.000-Inf)	0.996		
Fundus	1	1.000 (0.000-Inf)	1.000		
Gastric antrum	65	26698640.929 (0.000-Inf)	0.996		
Gastric corpus	13	33039305.878 (0.000-Inf)	0.996		
Others	2	20383228.678 (0.000-Inf)	0.996		
Surgical methods	96				
Distal gastrectomy	64	Reference			
Proximal gastrectomy	16	0.968 (0.487–1.925)	0.926		
Total gastrectomy	14	1.753 (0.904–3.398)	0.097		
Others	2	1.578 (0.382–6.522)	0.529		
AFP	78	1.009 (1.002–1.017)	**0.018**	1.015 (1.006–1.024)	**< 0.001**
CEA	78	1.005 (0.995–1.016)	0.313		
CA199	78	1.000 (0.999–1.002)	0.578		
CA153	77	1.010 (0.987–1.033)	0.406		
CA125	77	1.000 (0.994–1.007)	0.954		
H pylori infection	74				
( +)	37	Reference			
(−)	37	1.205 (0.699–2.076)	0.503		
IOD of BTG2	97	1.000 (0.986–1.015)	0.969		

qRT-PCR confirmed that high hsa_circ_0069382 expression upregulated BTG2 expression in HGC-27 and AGS cells (Additional file 1: Fig. S2K, L). The hsa_circ_0069382 overexpression plasmid was co-transfected with miR-15a-5p mimics, and we noted that overexpression of miR-15a-5p restored the effect of hsa_circ_0069382 for BTG2 (Additional file 1: Fig. S2K, L).

Western Blot results revealed that FAK expression in the LV3-miR group was higher than in the control group (LV3) (Fig. 6R, S). In contrast, its expression was lower in the LV5-circ group than in the control group (LV5) (Fig. 6R, S).

Transcriptional regulation in eukaryotes is a complex process involving multiple factors. To further explore the upstream regulatory mechanism of hsa_circ_0069382, transcription factors upstream of hsa_circ_0069382 were predicted using the DB toolkit and the Animal TFDB database. Then we obtained 58 common genes in both databases (Fig. 6T, Additional file 1: Table S4).

In addition to exploring the transcription factors of hsa_circ_0069382, we also found that hsa_circ_0069382 has the potential to encode proteins. CircRNA is known to be incapable of cap-dependent translation. However, recent studies have found that some circRNA with ORF bind to ribosomes and translate short peptides and proteins by IRES and m6A modification. Interestingly, we found that hsa_circ_0069382 had 19 IRESs and two ORFs, which were predicted by the IRESite database and ORF finder, respectively (Additional file 1: Tables S6, S7).

## Discussion

In this study, we identified a new circRNA, hsa_circ_0069382, using a circRNA chip and circBank database. Using the circRNA chip, nine paired samples of gastric cancer and adjacent tissues were evaluated. A total of 5396 circRNAs were detected, of which 212 were upregulated and 54 were downregulated. Hsa_circ_0069382, also known as hsa_circTBC1D19_011, is a member of the circRNA family. Both RNase R digestion and qRT-PCR confirmed its circular structure (Fig. 4J–L). The gene is located on chromosome 4, and is 673 bp long. It belongs to exon circular RNA. Our study found that hsa_circ_0069382 is expressed at low levels in gastric cancer cell lines and tissues. As shown in Graphical Abstracts, high hsa_circ_0069382 expression inhibited gastric cancer cell proliferation, invasion, and migration by downregulating miR-15a-5p and FAK, and upregulating BTG2. High miR-15a-5p expression partially restored the effect of high hsa_circ_0069382. MiR-15a-5p is highly evolutionarily conserved. Many studies have shown that miR-15a-5p is highly expressed in various cancers. MiR-15a-5p inhibits the expression of the target gene by binding to its downstream 3’-UTR to promote tumorigenesis [42, 43]. BTG2 is a downstream target molecule of miR-15a-5p, and many studies have shown that BTG2 regulates FAK expression in different cancers [40, 41]. Changes in hsa_circ_0069382 and miR-15a-5p affected the level of FAK in the hsa_circ_0069382 and miR-15a-5p overexpression groups. Our results demonstrated that hsa_circ_0069382 and miR-15a-5p are potential diagnostic biomarkers and therapeutic targets for gastric cancer. CircRNAs are thought significant molecules of the non-coding RNA and they function as miRNAs sponges [7]. circNRIP1, a attracted much attention circRNA, was found sponging miR-149-5p to regulate the expression of AKT1/mTOR, thereby promoting gastric cancer [24]. Additionally, circNRIP1 also regulates the development of ovarian cancer, renal cancer, nasopharyngeal carcinoma, and other malignant tumors through other ceRNA mechanisms [44–46]. In this study, we established that hsa_circ_0069382 regulates the expression of BTG2/FAK by sponging miR-15a-5p, thus affecting the proliferation, invasion, and migration of gastric cancer. Recent studies indicate that circRNAs can be used as a protein scaffold to directly regulate the expression of functional proteins [47, 48]. Most circRNAs are localized in the nucleus; they link the target proteins and regulate their expression through the Ago 2 protein [49]. Using FISH, we detected that hsa_circ_0069382 was expressed in both the nucleus and cytoplasm; however, it displayed stronger nuclear fluorescence (Fig. 4M). This suggested that in addition to sponging miR-15a-5p in the cytoplasm, it was possible for hsa_circ_0069382 to regulate potential nuclear proteins, thereby inhibiting gastric cancer.

Through bioinformatics analysis, we identified 58 potential transcription factors for hsa_circ_0069382 (Fig. 6T, Additional file 1: Table S4). Further research is needed to determine the nuclear role of hsa_circ_0069382 in the gastric cancer cells. Due to the lack of a 5'-cap structure and a 3'-A tail, circRNAs were considered not to translate proteins [50]. Recently, it was shown that a few circRNAs have been translated into functional short peptides and proteins directly [51]. Unlike the classical translation mechanism, circRNA translation requires an IRES or m6A methylation [52]. Thus, circRNAs with IRESs and ORFs may be translated into proteins or peptides. Using the IRESite and ORF finder database, we found that hsa_circ_0069382 had 19 IRESs (Additional file 1: Table S6) and two ORFs (Additional file 1: Table S7). These results suggested that hsa_circ_0069382 may code for a protein. However, whether hsa_circ_0069382 does so in gastric cancer, requires further studies.

MiR-15a-5p has been identified as an early found miRNA. Several studies have shown that miRNAs play different roles in diverse types of tumors. Yang et al. found that circZNF609 promotes the proliferation, metastasis, and stem cell of hepatocellular carcinoma by downregulating miR-15a-5p [53]. Guo et al. showed that lncRNA MEG8 promotes the proliferation of non-small cell lung cancer by downregulating miR-15a-5p [54]. These studies highlighted the tumor-suppressive effect exerted by miR-15a-5p. However, as mentioned earlier, miR-15a-5p has a tumor-promoting effect in cervical cancer and acute lymphoblastic leukemia [42, 43]. MiR-15a-5p expression may have spatial specificity, and it may play diverse roles in different tissues and cells. Our study confirmed that miR-15a-5p is highly expressed in gastric cancer tissues and cells, and promotes cancer by targeting the BTG2/FAK axis.

BTG2 belongs to the TOB/BTG gene family and has an anti-proliferative ability [55]. BTG2 is highly expressed in the stomach, intestine, spleen, pancreas, and other organs of the body. It was found that BTG2 inhibit the transformation from G1/S phase to G2/M phase and proliferation, promote apoptosis, and induce DNA repair to inhibit thymocyte expansion. It also affected the differentiation of nerve and hematopoietic cells [56]. Many studies have found that BTG2 is closely related to p53, p73, RB, and other tumor suppressor genes, it is downregulated in various tumors, such as gastric, laryngeal, and breast cancer [57–59]. Our study found that the expression of BTG2 was downregulated in gastric cancer tissues and cells, and that miR-15a-5p regulated its expression.

FAK is a tyrosine kinase involved in cancer cell invasion and metastasis [60]. It is regulated by a variety of signal transduction factors such as, cytokines, growth factors, integrins, and G protein-coupled receptors [61]. Yoon et al. reported that FAK also regulated the stemness and drug resistance of liver cancer stem cells by affecting the extracellular signal-regulated kinase 1/2 (ERK1/2) [62]. Our study found that FAK expression was lower in MKN-45, AGS, and HGC-27 cell line than in GES-1 cell line. Western blotting results showed that high miR-15a-5p expression increased FAK levels in AGS, while increased hsa_circ_0069382 expression had the opposite effect. Lentivirus transfection and western blotting demonstrated that miR-15a-5p promoted gastric cell invasion and migration by upregulating FAK, whereas hsa_circ_0069382 overexpression blocked this molecular axis. Low FAK expression in MKN-45, AGS, and HGC-27 cells should be investigated further.

In a word, our study showed that hsa_circ_0069382 regulated the expression of BTG2/FAK by sponging miR-15a-5p. However, there are some limitations in the study. First, FISH experiments showed that hsa_circ_0069382 was expressed in both the nucleus and cytoplasm of gastric cancer cells, and there seemed to be stronger fluorescence in the nucleus, but we only explored the mechanism of hsa_circ_0069382 in the cytoplasm of gastric cancer cells and did not explore its function in the nucleus. Second, we found that hsa_circ_0069382 had 19 IRES and 2 ORFs without verification on whether hsa_circ_0069382 encode the protein. In the future, we will further explore whether hsa_circ_0069382 plays a regulatory role in gastric cancer by encoding a micro-peptide protein.

In conclusion, our study showed that hsa_circ_0069382 sponged miR-15a-5p to regulate the expression of BTG2/FAK, affecting the proliferation, invasion, and migration of gastric cancer. Hsa_circ_0069382 and miR-15a-5p have potential as diagnostic markers and therapeutic targets for gastric cancer. The in-depth mechanism by which hsa_circ_0069382 regulates gastric cancer requires further research.

## Supplementary Information

**Additional file 1: Fig. S1** qRT-PCR examined the outcome of transfecting miR-15a-5p inhibitor in (**A**) HGC-27, (**B**) SGC-7901, and (**C**) AGS cells. qRT-PCR examined the outcome of transfecting miR-15a-5p mimics in (**D**) HGC-27, (**E**) SGC-7901, and (**F**) AGS cells. (**G**) Graphical representation the miR-15a-5p inhibitor effect on apoptosis of HGC-27 and AGS cells. (**H**) Graphical representation of the effect of miR-15a-5p inhibitor and mimics on AGS cell cycle. Luciferase reporter gene assays at (**I**) 24 h and (**J**) 48 h in miR-15a-5p and BTG2. TCGA data showed low levels of BTG2 in gastric cancer tissues of (**K**) paired and (**L**) unpaired samples. (**M**) Correlation between miR-15a-5p and BTG2 in gastric cancer in TCGA. (**N**) Correlation between miR- 15a-5p and FAK (PTK2) in gastric cancer in TCGA. (**O**) Immunohistochemical staining results from the Human Protein Atlas database showed that FAK was highly expressed in gastric cancer. **Fig. S2** (**A**) qRT-PCR for the expression of the homologous linear molecule TBC1D19 of hsa_circ_0069382 in gastric cancer cells. Luciferase reporter gene assays at (**B**) 24 h and (**C**) 48 h in miR-15a-5p and hsa_circ_0069382. qRT-PCR results of transfection shows the expression of hsa_circ_0069382 in (**D**) PEX-3 and hsa_circ_0069382 plasmid transfection group in HGC-27, (**E**) PEX-3 plasmid, hsa_circ_0069382 plasmid, and miR-15a-5p mimics with hsa_circ_0069382 plasmid transfection group in HGC-27 (**F**) hsa_circ_0069382 plasmid had no effect on TBC1D19 in HGC-27 (**G**) The expression of hsa_circ_0069382 in PEX-3 plasmid, hsa_circ_0069382 plasmid, and miR-15a-5p mimics with hsa_circ_0069382 plasmid transfection group in AGS (**H**) hsa_circ_0069382 plasmid had no effect on TBC1D19 in AGS. The expression of miR-15a-5p in PEX-3 plasmid, hsa_circ_0069382 plasmid, and miR-15a-5p mimics with hsa_circ_0069382plasmid transfection group in (**I**) HGC-27 and (**J**) AGS. The expression of BTG2 in PEX-3 plasmid, hsa_circ_0069382 plasmid, and miR-15a-5p mimics with hsa_circ_0069382 plasmid transfection group in (**K**) HGC-27 and (**L**) AGS. **Fig. S3** Lentivirus transfection and colony formation. The structure of (**A**) LV3 and (**B**) LV5. The lentivirus transfection results of (**C**) AGS and (**D**) SGC-7901, scale bar, 20μm. (**E**) Colony formation of AGS. (**F**), (**G**) Statistical chart of E. (**H**) (**I**) Statistical chart of C, D. **Table S1.** Target genes of miR-15a-5p. **Table S2.** The clinical features of 68 gastric cancer patients. **Table S3.** Prediction of binding sites between miR-15a-5p and circRNA. **Table S4.** Predicted upstream transcription factors of hsa_circ_0069382. **Table S5.** Primers used for qRT-PCR, inhibitor and mimic sequences, FISH probe sequences, lentivirus insertion sequences and antibodies. **Table S6.** Potential IRES for hsa_circ_0069382. **Table S7.** Potential ORFs for hsa_circ_0069382.

## Data Availability

The original contributions presented in the study are included in the article/Additional file Material.
