# Supplementary material for: A novel circRNA, hsa_circ_0069382, regulates gastric cancer progression
Source: Cancer Cell Int. 2023 Feb 25;23:35. doi: 10.1186/s12935-023-02871-4 (PMC9960672; doi:10.1186/s12935-023-02871-4)
Supplement: Supplementary file 1 — Additional file 1: Fig. S1 qRT-PCR examined the outcome of transfecting miR-15a-5p inhibitor in (A) HGC-27, (B) SGC-7901, and (C) AGS cells. qRT-PCR examined the outcome of transfecting miR-15a-5p mimics in (D) HGC-27, (E) SGC-7901, and (F) AGS cells. (G) Graphical representation the miR-15a-5p inhibitor effect on apoptosis of HGC-27 and AGS cells. (H) Graphical representation of the effect of miR-15a-5p inhibitor and mimics on AGS cell cycle. Luciferase reporter gene assays at (I) 24 h and (J) 48 h in miR-15a-5p and BTG2. TCGA data showed low levels of BTG2 in gastric cancer tissues of (K) paired and (L) unpaired samples. (M) Correlation between miR-15a-5p and BTG2 in gastric cancer in TCGA. (N) Correlation between miR- 15a-5p and FAK (PTK2) in gastric cancer in TCGA. (O) Immunohistochemical staining results from the Human Protein Atlas database showed that FAK was highly expressed in gastric cancer. Fig. S2 (A) qRT-PCR for the expression of the homologous linear molecule TBC1D19 of hsa_circ_0069382 in gastric cancer cells. Luciferase reporter gene assays at (B) 24 h and (C) 48 h in miR-15a-5p and hsa_circ_0069382. qRT-PCR results of transfection shows the expression of hsa_circ_0069382 in (D) PEX-3 and hsa_circ_0069382 plasmid transfection group in HGC-27, (E) PEX-3 plasmid, hsa_circ_0069382 plasmid, and miR-15a-5p mimics with hsa_circ_0069382 plasmid transfection group in HGC-27 (F) hsa_circ_0069382 plasmid had no effect on TBC1D19 in HGC-27 (G) The expression of hsa_circ_0069382 in PEX-3 plasmid, hsa_circ_0069382 plasmid, and miR-15a-5p mimics with hsa_circ_0069382 plasmid transfection group in AGS (H) hsa_circ_0069382 plasmid had no effect on TBC1D19 in AGS. The expression of miR-15a-5p in PEX-3 plasmid, hsa_circ_0069382 plasmid, and miR-15a-5p mimics with hsa_circ_0069382plasmid transfection group in (I) HGC-27 and (J) AGS. The expression of BTG2 in PEX-3 plasmid, hsa_circ_0069382 plasmid, and miR-15a-5p mimics with hsa_circ_0069382 plasmid transfection group [file 12935_2023_2871_MOESM1_ESM.pdf]

Figure S1

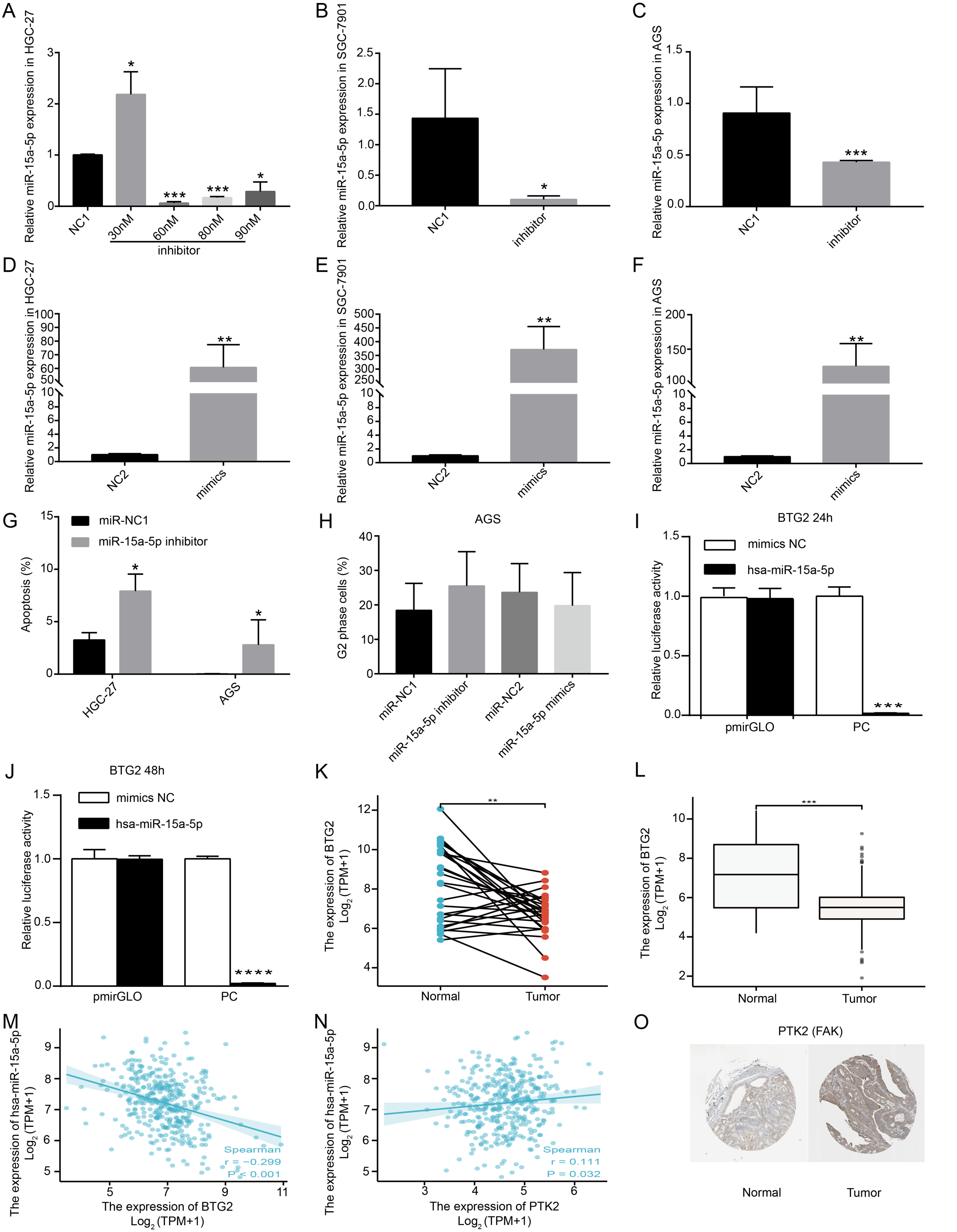

A
Relative miR-15a-5p expression in HGC-27
NC1
30nM
60nM
80nM
90nM
inhibitor
B
Relative miR-15a-5p expression in SGC-7901
NC1
inhibitor
C
Relative miR-15a-5p expression in AGS
NC1
inhibitor
D
Relative miR-15a-5p expression in HGC-27
NC2
mimics
E
Relative miR-15a-5p expression in SGC-7901
NC2
mimics
F
Relative miR-15a-5p expression in AGS
NC2
mimics
G
miR-NC1
miR-15a-5p inhibitor
Apoptosis (%)
HGC-27
AGS
H
AGS
G2 phase cells (%)
miR-NC1
miR-15a-5p inhibitor
miR-NC2
miR-15a-5p mimics
I
BTG2 24h
mimics NC
hsa-miR-15a-5p
Relative luciferase activity
pmirGLO
PC
J
BTG2 48h
mimics NC
hsa-miR-15a-5p
Relative luciferase activity
pmirGLO
PC
K
The expression of BTG2
Log2 (TPM+1)
Normal
Tumor
L
The expression of BTG2
Log2 (TPM+1)
Normal
Tumor
M
The expression of hsa-miR-15a-5p
Log2 (TPM+1)
Spearman
r = −0.299
P < 0.001
The expression of BTG2
Log2 (TPM+1)
N
The expression of hsa-miR-15a-5p
Log2 (TPM+1)
Spearman
r = 0.111
P = 0.032
The expression of PTK2
Log2 (TPM+1)
O
PTK2 (FAK)
Normal
Tumor

Figure S2

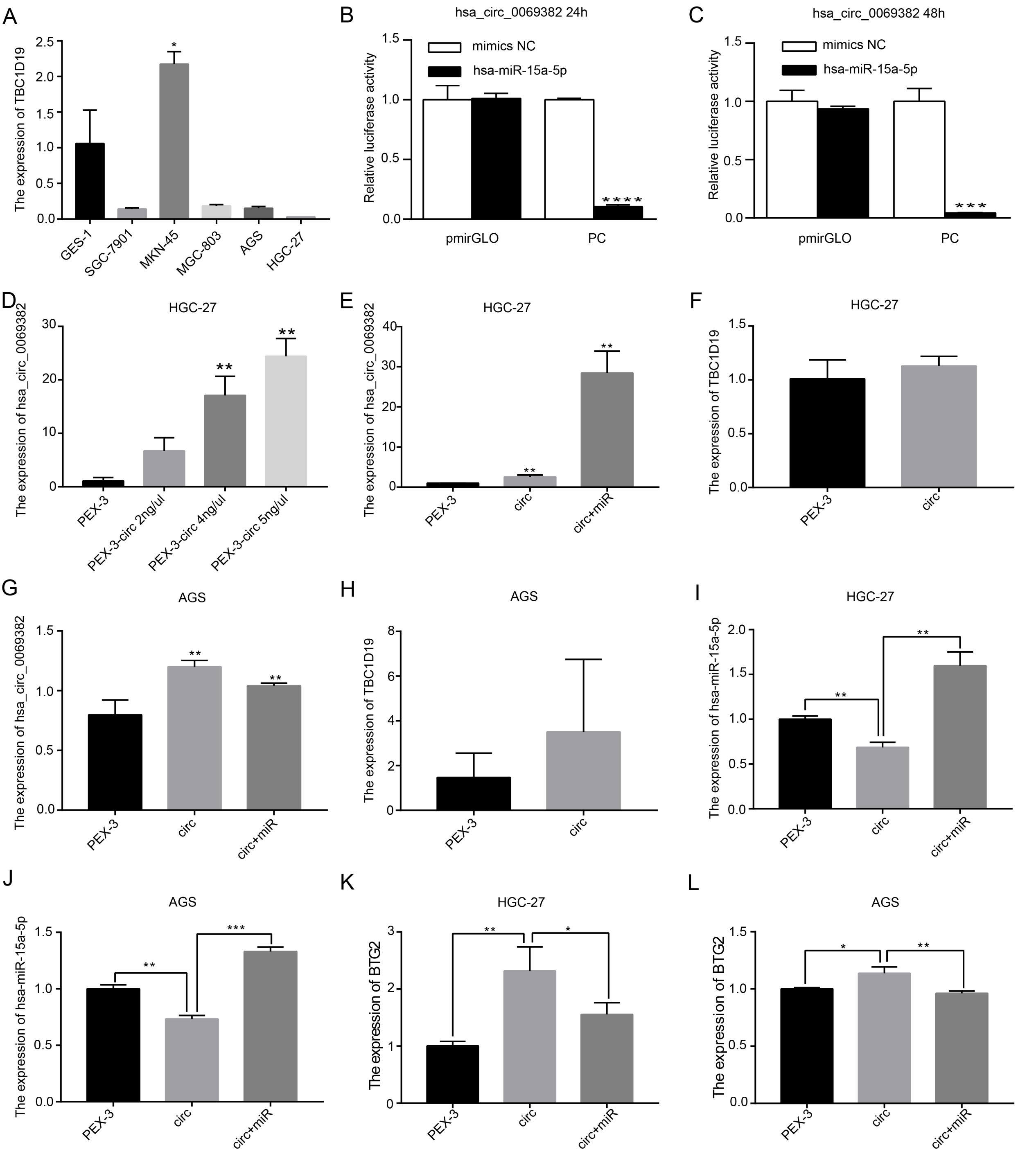

A
The expression of TBC1D19
GES-1
SGC-7901
MKN-45
MGC-803
AGS
HGC-27
B
hsa_circ_0069382 24h
Relative luciferase activity
mimics NC
hsa-miR-15a-5p
pmirGLO
PC
C
hsa_circ_0069382 48h
Relative luciferase activity
mimics NC
hsa-miR-15a-5p
pmirGLO
PC
D
HGC-27
The expression of hsa_circ_0069382
PEX-3
PEX-3-circ 2ng/ul
PEX-3-circ 4ng/ul
PEX-3-circ 5ng/ul
E
HGC-27
The expression of hsa_circ_0069382
PEX-3
circ
circ+miR
F
HGC-27
The expression of TBC1D19
PEX-3
circ
G
AGS
The expression of hsa_circ_0069382
PEX-3
circ
circ+miR
H
AGS
The expression of TBC1D19
PEX-3
circ
I
HGC-27
The expression of hsa-miR-15a-5p
PEX-3
circ
circ+miR
J
AGS
The expression of hsa-miR-15a-5p
PEX-3
circ
circ+miR
K
HGC-27
The expression of BTG2
PEX-3
circ
circ+miR
L
AGS
The expression of BTG2
PEX-3
circ
circ+miR

Figure S3

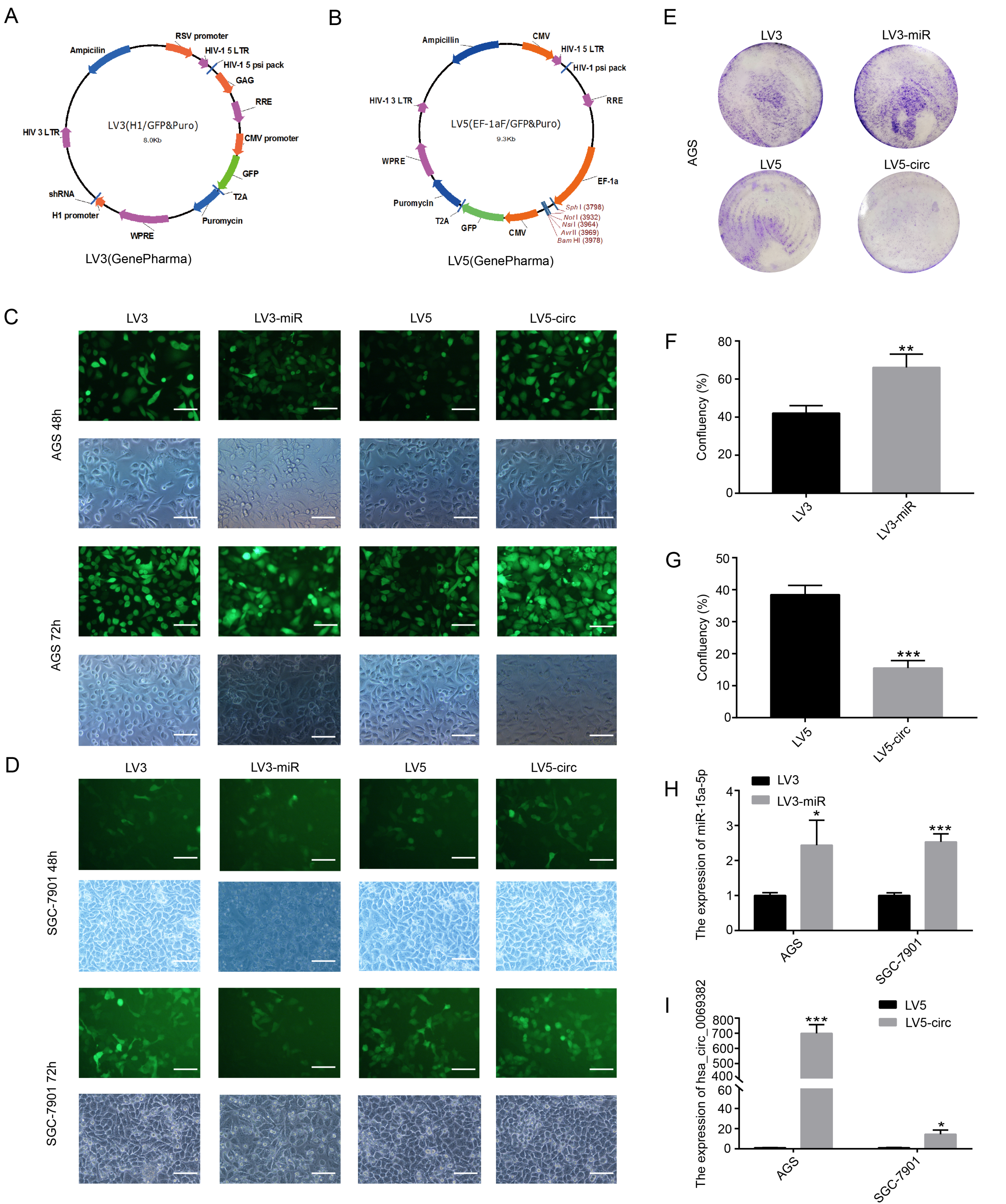

A
Ampicilin
RSV promoter
HIV-1 5 LTR
HIV-1 5 psi pack
GAG
RRE
CMV promoter
GFP
T2A
Puromycin
WPRE
H1 promoter
shRNA
HIV 3 LTR
LV3(H1/GFP&Puro)
8.0Kb
LV3(GenePharma)
B
Ampicillin
CMV
HIV-1 5 LTR
HIV-1 psi pack
RRE
EF-1a
Sph I (3798)
Not I (3932)
Nsi I (3964)
Avr II (3969)
Bam HI (3978)
CMV
GFP
T2A
Puromycin
WPRE
HIV-1 3 LTR
LV5(EF-1aF/GFP&Puro)
9.3Kb
LV5(GenePharma)
E
AGS
LV3
LV3-miR
LV5
LV5-circ
C
LV3
LV3-miR
LV5
LV5-circ
AGS 48h
AGS 72h
D
LV3
LV3-miR
LV5
LV5-circ
SGC-7901 48h
SGC-7901 72h
F
Confluency (%)
0
20
40
60
80
**
LV3
LV3-miR
G
Confluency (%)
0
10
20
30
40
50
***
LV5
LV5-circ
H
The expression of miR-15a-5p
LV3
LV3-miR
0
1
2
3
4
*
***
AGS
SGC-7901
I
The expression of hsa_circ_0069382
LV5
LV5-circ
0
20
40
60
400
500
600
700
800
***
*
AGS
SGC-7901

Table S1 Target genes of miR-15a-5p								
gene name	fold change	p-value	gene name	fold change	p-value	gene name	fold change	p-value
CCNE1	10.21	2.10E-12	DENND6A	1.15	8.50E-03	ZBTB10	1.48	3.80E-02
SPRED1	1.71	4.20E-11	CPEB3	0.71	2.20E-05	SALL1	0.96	2.70E-01
WNT3A	0.65	0.19	PDCD4	0.67	5.60E-04	NR2C2	1.4	8.90E-06
LSM11	1.23	3.10E-05	PLEKHA1	1.48	3.30E-05	CDK6	4.44	1.10E-10
WEE1	1.18	4.60E-02	BZW1	1.45	1.60E-09	EN2	16.42	2.50E-04
CDCA4	2.02	2.00E-13	C16orf72	1.12	3.90E-02	OCRL	1.08	8.40E-02
SLC9A6	1.14	7.30E-02	PIM1	0.45	3.10E-10	KIF3B	1.67	1.50E-11
SNCG	0.47	1.80E-09	RARB	0.74	1.30E-02	ZNF367	3.55	2.40E-27
UBE2V1	1.57	3.90E-09	DDX3Y	0.64	3.30E-01	ANKRD13B	2.93	1.70E-14
FGF7	0.85	1.20E-01	ZCCHC3	1.48	1.40E-04	TASP1	1.94	8.70E-18
CDC42SE2	1.37	1.00E-05	DMTF1	1.65	7.80E-08	PPM1A	0.76	4.00E-05
C2orf42	1.18	5.50E-04	RAB11FIP2	1	8.40E-01	CDV3	1.23	1.30E-04
PURA	0.89	6.10E-02	RAD23B	1.3	1.60E-05	NOTCH2	1.16	3.50E-02
CDC37L1	0.98	5.00E-01	RTN4	0.92	2.30E-01	PTPRJ	1.92	2.20E-13
C1orf21	0.65	8.60E-04	MIB1	1.71	1.50E-08	MTFR1L	0.63	1.50E-11
CCND1	2.07	8.00E-08	AP2B1	1.95	3.30E-16	RASSF5	0.69	4.10E-01
BTG2	0.23	1.70E-14	ASH1L	1.2	7.10E-03	CSDE1	0.82	7.10E-03
AXIN2	2.64	3.10E-01	TSPYL2	0.39	7.70E-06	SIK1	0.67	2.50E-02
SMAD7	1.05	8.80E-01	GPATCH8	1.42	5.90E-09	PPP1R11	1.01	7.90E-01
N4BP1	0.92	7.00E-01	FKBP1A	1.56	4.20E-11	ZFHX4	0.3	1.90E-06
CACUL1	1.49	1.70E-11	RBBP6	1.32	2.60E-07	UBE4A	1.35	1.40E-03
CCND2	1.15	4.70E-01	KANK1	0.69	1.00E-02	FBXL20	1.34	1.80E-03
WIPI2	1.28	4.90E-06	E2F3	3.2	3.30E-37	KPNA1	1.35	2.70E-10
TLK1	1.12	6.20E-03	ACVR2A	1.01	7.20E-01	PRRC2C	2.07	2.50E-27
ATG9A	1.17	1.40E-02	LURAP1L	1.66	3.10E-04	AMOTL1	0.54	1.90E-02
KIF23	5.62	1.20E-37	RAP2C	1.61	2.00E-12	HOXA3	1.02	4.80E-01
HMGA2	19.45	3.30E-14	HOXA10	15.3	4.30E-20	RBPJ	1.42	4.90E-10
MOB4	1.08	8.80E-02	BCL7A	2.16	3.80E-15	MLLT6	1.34	6.30E-03
VEGFA	2.39	7.50E-09	APP	1.24	2.10E-03	CTDSPL	1.23	1.80E-02
MAFK	1.31	6.50E-03	BCL2	0.64	1.60E-05	TLE4	0.59	2.80E-08
PEX13	1.32	6.10E-07	PDIK1L	1.75	8.00E-12	MTMR4	1.45	2.10E-06
SHOC2	1.21	3.80E-03	YAP1	1.2	1.30E-02	SMURF1	1.67	1.80E-07
RNF138	1.56	1.20E-08	CUL2	1.49	9.10E-11	AMOT	2.04	1.30E-02
CAPZA2	1.24	1.40E-01	PAG1	1.66	8.60E-06	WNK3	0.7	1.20E-01
RUNX1T1	0.74	1.20E-01	PPP6C	1.22	0.000.14	ATP13A3	2.78	4.10E-25
PLAG1	1.88	3.60E-01	YTHDC1	1.19	2.50E-05	KPNA3	1.49	1.30E-09
AMER1	1.15	3.50E-01	CREBRF	0.96	5.80E-01	CREBL2	1.04	9.60E-01
CRKL	1.42	2.10E-07	PISD	1.46	4.40E-10	STXBP3	1.43	2.70E-03
CHEK1	3.61	7.80E-29	JARID2	1.07	5.80E-01	PPP6R3	1.47	1.20E-08
EM189-UBE2	1.16	1.10E-01	EFNB2	1.35	9.10E-03	TAOK1	1.5	1.10E-08
RPS6KA3	1.59	1.30E-07	NUP50	1.6	1.90E-12	PTPRD	0.84	5.00E-03
FGF2	0.36	5.10E-06	YWHAQ	1.2	6.10E-04	TBL1XR1	2.17	3.00E-18
FASN	2	3.20E-09	PRKAR2A	1.24	8.10E-03	UBR3	1.15	1.90E-03
HSPA4L	1.02	8.70E-01	CBFA2T3	0.68	1.30E-04	ACTR1A	0.88	6.50E-03
UBE2Q1	1.51	1.30E-13	ZBTB33	2.09	7.50E-23	FAM122B	2.59	1.40E-17
ACTR2	1.57	1.80E-16	B4GALT1	1.44	6.90E-05	CHIC1	0.83	3.60E-02
CCNT2	1.65	3.10E-10	PNISR	1.22	1.70E-02	RIMS3	0.58	1.80E-05
TBPL1	1.25	9.60E-04	TSC22D2	0.82	3.80E-02	LRRFIP2	0.96	4.40E-01
YWHAH	1.22	9.00E-05	MAP2K3	1.05	2.60E-01	MKX	0.55	4.70E-02
E2F7	5.47	1.70E-29	PANK1	1.22	7.80E-03	MYO5A	1.22	3.80E-01
ENTPD7	1.03	2.40E-05	BACE1	1.08	5.90E-01	ZNF704	1.03	3.00E-01
GABARAPL1	0.54	1.30E-07	CRIM1	1.12	9.90E-02	TRAM1	1.88	1.70E-13
PAFAH1B1	0.96	2.80E-01	SLC25A22	2.02	3.20E-12	USP31	1.75	5.20E-20
RECK	0.52	5.10E-06	HOXC8	17.35	9.50E-27	NAA25	2.28	2.40E-28
CPEB2	0.68	1.00E-06	SOX5	0.55	1.20E-04	CD2AP	1.72	9.90E-09
SNX16	1.14	3.40E-02	EZH1	0.72	7.80E-03	OGT	2.08	3.10E-12
ZNF449	1.43	7.10E-08	SIDT2	0.57	1.70E-08	DDX3X	1.16	2.50E-02
AGO4	1.05	5.10E-01	SERBP1	1.44	1.70E-11	ATG14	1.14	5.30E-03
STRADB	0.75	1.30E-01	RET	1.39	1.10E-01	SREK1	1.63	1.50E-11
CDK17	1.27	8.80E-04	AFF4	1.04	7.90E-01	CDS2	0.9	7.10E-01
BDNF	2.13	1.80E-03	HMBOX1	1.23	1.50E-02	USP15	1.37	1.00E-05
CARM1	1.36	5.20E-05	TMEM135	1.43	3.80E-06	ETNK1	1.55	2.10E-05
LUZP1	1.08	3.60E-02	PIK3R1	0.95	9.40E-01	SRPK1	2.21	9.60E-23
CDC27	1.67	3.90E-16	PSAT1	2.96	3.30E-07	NUFIP2	1.94	2.90E-23
HMGA1	2.24	1.80E-14	CLCN3	1.43	2.80E-04	SYPL1	1.26	8.90E-03
CCNYL1	0.96	4.30E-01	CAMSAP1	1.13	3.50E-04	DICER1	1.3	1.40E-04
USP3	1.46	2.30E-08	LAMC1	1.16	4.80E-03	CADM1	1.07	2.20E-02

Table S2 The clinical features of 68 gastric cancer patients					
NO.	Gender	Age	differentiation	Clinical TNM stage	Lauren classification
1	male	63	poorly	T3N3aMx	diffuse-type
2	male	90	poorly	T4aN3Mx	diffuse-type
3	male	64	moderate-poorly	T3N2Mx	diffuse-type+ intestinal-type
4	male	53	moderate-poorly	T4N0Mx	diffuse-type+ intestinal-type
5	female	62	unknow	T4N0Mx	
6	male	76	poorly	T3N0Mx	
7	female	65	moderate-poorly	T3N0Mx	intestinal-type
8	male	76	poorly	T3N3aMx	intestinal-type +diffuse-type
9	male	70	poorly	T3N0Mx	diffuse-type
10	female	69	moderate-poorly	T1bN0Mx	intestinal-type
11	male	40	poorly	T3N3aMx	diffuse-type
12	male	51	moderate-poorly	T3N3aMx	
13	female	74	moderate-poorly	T4aN2Mx	mixed-type
14	female	45	poorly	T1aN0Mx	diffuse-type
15	female	66	moderate-poorly	T3N1Mx	diffuse-type
16	female	52	poorly	T3N0Mx	diffuse-type
17	female	36	poorly	T2N3aMx	diffuse-type
18	female	56	moderate-poorly	T3N2Mx	mixed-type
19	female	82	moderate-poorly	T4N1Mx	
20	female	73	moderate-poorly	T3N2Mx	intestinal-type
21	male	71	moderate-poorly	T2N2Mx	intestinal-type+diffuse-type
22	male	67	moderate-poorly	T4aN1Mx	mixed-type
23	male	64	moderate-poorly	T4aN2Mx	mixed-type
24	male	73	moderate-poorly	T3N3aMx	diffuse-type
25	male	53	poorly	T4aN3bMx	diffuse-type
26	male	56	moderate-poorly	T4aN3aMx	mixed-type
27	male	55	moderate-poorly	T4bN2Mx	diffuse-type
28	male	80	poorly	T2N0Mx	diffuse-type
29	male	45	moderate	T1bN0Mx	intestinal-type
30	male	70	moderate-poorly	T3N3aMx	diffuse-type
31	male	84	poorly		diffuse-typegastric cancer
32	male	64	unknow	T3N0Mx	
33	male	80	moderate-poorly	T3N3aMx	diffuse-type+ intestinal-type
34	male	75	moderate-poorly	T2N1Mx	intestinal-type +diffuse-type
35	male	54	poorly	T4aN3Mx	intestinal-type +diffuse-type
36	male	55	poorly	T3N2Mx	mixed-type
37	male	80	moderate-poorly	T1aN0Mx	intestinal-type+diffuse-type
38	male	71	moderate-poorly	T4aN3aMx	intestinal-type+diffuse-type
39	male	57	poorly	T4aN3Mx	
40	male	65	moderate-poorly	T3N0Mx	
41	female	69	moderate-poorly	T4aN2Mx	
42	male	57	moderate-poorly	T4N3Mx	
43	male	70	moderate-poorly	T4N2Mx	
44	male	72	moderate	T4aN0Mx	
45	female	56	poorly	T4N3Mx	
46	female	64	unknow	T3N2Mx	
47	male	57	moderate	T2N0Mx	
48	male	64	poorly	T4N2Mx	
49	male	58	unknow	T4aN2bMx	
50	male	69	poorly	T3N2Mx	
51	female	69	poorly	T4aN3bMx	
52	male	78	moderate-poorly	T4N2M1	
53	male	64	moderate-poorly	T3N2Mx	intestinal-type
54	female	53	moderate-poorly	T3N0Mx	mixed-type

55	male	57	moderate-poorly	T1bN0Mx	mixed-type
56	male	53	moderate-poorly	T4aN2Mx	mixed-type
57	male	66	moderate	T1bN1Mx	intestinal-type
58	male	72	moderate	T2N3aMx	intestinal-type
59	male	68	moderate-poorly	T3N1Mx	mixed-type
60	female	52	poorly	T3N0Mx	diffuse-type
61	female	31	poorly	T1bN0Mx	diffuse-type
62	male	76	poorly	T3N3aMx	diffuse-type
63	male	68	moderate-poorly	T3N3aMx	mixed-type
64	male	44	poorly	T2N2Mx	diffuse-type
65	male	52	poorly	T4aN2Mx	diffuse-type
66	male	71	moderate-poorly	T3N0Mx	mixed-type
67	male	47	moderate-poorly	T3N2Mx	mixed-type
68	male	76	moderate-poorly	T2N1Mx	mixed-type

Table S3 Prediction of binding sites between miR-15a-5p and circRNA					
**circRNA**	**Length**	**MFE**	***p* value**	**Position**	**binding site**
hsa_circ_0069382	673	-25.3 kcal/mol	undefined	490	Target 5' A A AG A C 3'<br>GCAGA CA AU GUGCUGCUG<br>UGUUU GU UA CACGACGAU<br>miRNA 3'G G AA 5'
hsa_circ_0055954	1375	-20.7 kcal/mol	undefined	442	target 5' G CU UUUU G 3'<br>ACGA ACU UGCUGCUG<br>UGUU UGG ACGACGAU<br>miRNA 3' G UAAUAC 5'
hsa_circ_0006278	400	-21.2 kcal/mol	undefined	12	target 5' C C C G 3'<br>UAA CCAU UGCUGCUA<br>GUU GGUA ACGACGAU<br>miRNA 3' GU U AUAC 5'

Table S4 Predicted upstream transcription factors of hsa_circ_0069382		
DB toolkit	Animal TFDB	intersection (58)
GRHL3	ZNF143	SMARCA4
SALL4	ZNF384	KDM1A
SOX2	MAZ	POU5F1
SMAD1	SMARCA4	CDX2
CBX3	KDM1A	BMI1
PTTG1	ZNF92	FOXM1
NKX3-1	FOXP1	EP300
BIRA	NOTCH1	TRIM28
ZNF649	MAFF	FOXA1
TRIM28	POU5F1	NKX3-1
DDX20	EHF	MED1
H3F3B	SP1	BRD4
DPF1	CDX2	ESR1
CHD7	BMI1	MYC
BCL11A	HLTF	FOXJ2
ZC3H8	ZNF148	RXRA
IRF9	ZNF281	AR
OTX2	ZNF263	TRIM24
SMAD3	TBX10	EWSR1
ZNF823	TAF1	SPI1
ZNF586	FOXM1	RELA
SETDB1	PRDM1	SETDB1
POU5F1	CTBP2	GATA1
GATA4	EP300	CBX3
HINFP	IRF1	POLR2A
ATF4	FOXF1	ATF2
ZNF490	NR2C2	NANOG
SMAD2	ZFHX3	TCF7L2
AR	TRIM28	ATF3
SUMO2	REST	TEAD4
ZNF765	SOX18	CBX1
ERCC6	FOXA2	HOXB13
ZNF318	HOXB9	ERG
ID3	C17orf96	LMNB1
ZNF675	RFX3	SMAD1
ZNF585B	DBP	BCL11A
GATA1	TRIM63	SOX2
FOXO1	NFIC	SMAD3
DIDO1	ARNT	LEF1
DEK	NFIL3	OTX2
NANOG	POU3F2	GRHL3
BRD4	GATA3	GATA4
NUP98-HOXA	RREB1	USF2
CHAMP1	EZH2	NR4A1
TEAD4	NFE2L2	SALL4
MEF2D	TP53	IRF9
ZNF24	STAT1	SUMO2
ZNF324	BACH2	CHD7
GATA6	STAT3	ELF1
ZNF793	MAFK	ZC3H8
ZNF175	BRCA1	FOXH1
PBX3	MAF	FOXO1
PRDM14	SRF	GATA6
NR4A1	MEF2C	PBX3
NFRKB	FOXA1	FEZF1
ESR1	NKX3-1	NIPBL

RELA	NKX2-3	PIAS1
KDM1A	MED1	BACH1
BACH1	SP4	
FEZF1	BRD4	
CUX1	ZNF219	
ZNF83	PROP1	
NIPBL	SOX17	
ZNF486	ESR1	
NKRF	BHLHE40	
ZNF248	MYC	
SSU72	NFE2	
ZNF182	FOXJ2	
NBN	SP2	
ARID1A	BRD2	
RAD51	SUZ12	
LMNB1	STAT2	
EP300	RXRA	
ELF1	JUND	
FOXH1	YY1	
ERG	AR	
LEF1	MAFG	
MED1	VDR	
PIAS1	KDM5B	
RXRA	NR2F2	
SPI1	PURA	
ZNF280A	ELK1	
CDX2	CBFB	
MTA2	FLI1	
ZNF506	JUN	
ZNF84	MECOM	
FOXA1	TRIM24	
POLR2A	FOXD3	
ATF3	TEAD1	
TRIM24	GTF3C2	
POLR2H	EWSR1	
HMBOX1	PBX1	
ATF2	CREB1	
FOXM1	NPAT	
ZNF580	GTF2I	
E2F8	KLF1	
ZNF644	STAT2:STAT1	
HOXB13	SPI1	
FOXJ2	FOXQ1	
USF2	RELA	
TCF7L2	SETDB1	
ZNF589	HNF1A	
BMI1	SP7	
MYC	KLF5	
SMARCE1	PGR	
ZIM3	RARA	
EWSR1	KLF4	
SCRT1	LEF1:TCF1	
CBX1	GATA1	
ZNF146	CBX3	
ERCC2	IRF2	
SMARCA4	FOXK1	
SMAD2/3	SIN3A	
	TBP	

	ZNF250	
	HOXA13	
	LIN54	
	SP3	
	AIRE	
	SMAD4	
	POLR2A	
	HOXA7	
	BCL6	
	HNF4A	
	HEY1	
	HSF1	
	ATF2	
	NANOG	
	HLF	
	TAL1	
	RXRG	
	ZNF274	
	TCF7L2	
	KLF11	
	LCOR	
	IRF4	
	PML	
	GATA2	
	RUNX1	
	HDAC2	
	SMAD2:SMAD3:SMAD4	
	POU1F1	
	ATF3	
	WHSC1	
	FOXC1	
	STAT6	
	ARRB1	
	NFATC1	
	ETS1	
	FOXO3	
	TCF4	
	POLR3A	
	PPARD/PPARG	
	GABPA	
	PLAG1	
	RUNX2	
	TP73	
	TEAD4	
	CEBPG	
	SOX9	
	E2F7	
	ZBTB7B	
	PPARG:RXRA	
	NR2F1	
	COUP-TF:HNF4	
	EZH1	
	FOXF2	
	ARID3A	
	CEBPA	
	FUBP1	
	EPAS1	
	CBX1	

	E2F4	
	REX1	
	DDX5	
	IRF3	
	HOXB13	
	NR3C1	
	E4F1	
	FOXA3	
	PATZ1	
	FOXP2	
	VDR:CAR:PXR	
	TCF12	
	IRF5	
	HNF1B	
	CENPA	
	EGR1	
	ERG	
	TEAD3	
	NCOR1	
	SOX13	
	KLF7	
	POU2F3	
	GLYR1	
	LMNB1	
	PPARG	
	TCF3	
	VEZF1	
	SREBF1	
	NR6A1	
	POU4F3	
	POU4F1	
	ELF2	
	RBL2	
	ZNF740	
	MZF1	
	NFE2L1	
	KLF15	
	SMAD1	
	ZIC1	
	FOXI1	
	BRD3	
	TFAP2D	
	MAX	
	BCL11A	
	SOX2	
	OTP	
	SPIC	
	NR5A2	
	GMEB2	
	CHD1	
	BCOR	
	MITF	
	FOXJ3	
	RNF2	
	BARX2	
	FOXL1	
	BTAF1	
	NFYB	

	SMAD3	
	ARNT2	
	GLI1	
	IRF7	
	LEF1	
	RFX7	
	CDK8	
	LHX2	
	ETV4	
	MEF2A	
	POU2F1	
	ZFP42	
	BDP1	
	SOX14	
	RFX5	
	RUNX3	
	ZFY	
	PCGF2	
	ZBTB33	
	NANOGP1	
	TBR1	
	ZBTB17	
	CPEB1	
	RFX2	
	OTX2	
	HMGA2	
	ELF5	
	RARB	
	ZNF713	
	SREBF2	
	STAT5B	
	MTA3	
	GRHL3	
	MYOD1	
	ZIC2	
	KAT8	
	HOXA11	
	HOXC10	
	CREBBP	
	LTF	
	SUMO2/SUMO3	
	USF1	
	NKX6-1	
	NFYA	
	HMG20B	
	NR1D1	
	NKX2-2	
	FOXP4	
	GATA4	
	E2F1	
	USF2	
	NR4A1	
	TBX2	
	ZNF410	
	ETS2	
	44531	
	CREM	
	SALL4	

	ZNF784	
	ZNF202	
	TBX5	
	CEBPD	
	HOXA1	
	PDX1	
	ENSG00000234254	
	FOSL1	
	TBL1XR1	
	CDX4	
	SPIB	
	TAF3	
	NR1H4	
	SMARCC1	
	HOXD13	
	IRF8	
	PRAME	
	IRF9	
	TGIF2LY	
	CDX1	
	L3MBTL2	
	NCOR2	
	GTF2A1:GTF2A2	
	SUMO2	
	POU3F3	
	CHD7	
	FOXO6	
	OLIG2	
	PAX5	
	PXR:RXR	
	RUNX1T1	
	EOMES	
	ZNF691	
	MEIS1	
	ELL2	
	MYB	
	CEBPB	
	HOXD4	
	FOS	
	HDAC3	
	TGIF2LX	
	NR4A2	
	PPARA	
	NF1A	
	HIF1A	
	ASCL2	
	LMO2	
	LYL1	
	MXI1	
	SAP30	
	STAT5A	
	NR1D2	
	RARG	
	SRC	
	CDK9	
	EGR4	
	ZKSCAN1	
	KLF3	

	ICE1	
	ELF1	
	POU6F2	
	EBF1	
	MYBL2	
	RAC3	
	HOXD10	
	FOXP3	
	ICE2	
	HOXA9	
	IKZF1	
	DMRTA2	
	TBX4	
	CTCF	
	HES4	
	AL844527	
	PPARGC1A	
	TBX22	
	POU6F1	
	PAX3	
	T	
	NR3C2	
	CDC5L	
	HOXD9	
	ZC3H8	
	FOXH1	
	NKX6-3	
	FOXO1	
	TAL1:TCF3	
	GATA6	
	ETV1	
	SMAD2/SMAD3	
	ZNF280D	
	RXRB	
	TFAP4	
	NR2E3	
	GLI2	
	SRY	
	NR2C1	
	SMARCC2	
	TEF	
	BCL3	
	MEIS1B:HOXA9	
	NFYC	
	PBX3	
	CCNT2	
	NFATC2	
	GSX2	
	PRDM16	
	NFATC3	
	ORC1	
	FEZF1	
	NKX2-1	
	ZNF35	
	RBPJ	
	PHOX2A	
	NIPBL	
	HOXB1	

	VTN	
	ISL1	
	SOX15	
	HMGA1	
	STAG1	
	NRF1	
	RFX4	
	PIAS1	
	FOXO4	
	ELK3	
	HOXD8	
	MEOX2	
	DMRTA1	
	TBX15	
	HCFC1	
	HOXA10	
	RORA	
	TCF7	
	HLX	
	OLIG3	
	HOXC11	
	MAFB	
	TLX1	
	E2F6	
	ESRRG	
	ETV6	
	NEUROD1	
	NR1I2	
	NEUROG3	
	SOX1	
	ISGF3	
	BACH1	
	CBX4	
	NF1	
	NFAT5	
	FOXN3	
	HOXA2	
	ESRRA	
	LRH1	
	DMRT3	
	NFIB	
	MSX2	
	PRRX2	
	LIN9	
	RB1	
	ONECUT1	
	GTF2B	
	GATA5	
	TCF7L1	
	RAD21	
	CREB5	
	PRKDC	

**Table S5 Primers used for qRT-PCR**	
miR-15a-5p-F	CGTAGCAGCACATAATGGTTTGTG
miR-15a-5p-R	Universal primer (Taraka, 638313)
U6-F	GGAACGATACAGAGAAGATTAGC
U6-R	TGGAACGCTTCACGAATTTGCG
GAPDH-F	ACAACTTTGGTATCGTGGAAGG
GAPDH-R	GCCATCACGCCACAGTTTC
BTG2-F	CATCATCAGCAGGGTGGC
BTG2-R	CCCAATGCGGTAGGACAC
hsa circ 0069382-F	CACTGAGAGCTGAATTGTGGG
hsa circ 0069382-R	TTAAGATGTTCAGGAGGTGCAG
hsa circ 0002319-F	CATTTGGCCAATCATGTGCGA
hsa circ 0002319-R	GACCTTTGTTGGTTTCTGCCC
hsa circ 0004206-F	GCTTTCTGGGGAGTGAACAGT
hsa circ 0004206-R	ACTCTGGGTGTGTTTCATCCTC
hsa circ 0055954-F	AGAGAAACCCAAATCAGCCAT
hsa circ 0055954-R	GGGTGGCAGAATGAACCAA
hsa circ 0006278-F	TCTAAGTGGGAATCATTTCAGGTC
hsa circ 0006278-R	AGGTTATGGAAGTGGATCCTTCC
TBC1D19-F	TGGGCCAGTCTTCAGAGACC
TBC1D19-R	GGTGCAGCAGGATGACTAGG
**inhibitor and mimics sequences**	
miR-15a-5p inhibitor	CACAAACCAUUAUGUGCUGCUA
inhibitor NC	CAGUACUUUUGUGUAGUACAA
miR-15a-5p mimics sense	UAGCAGCACAUAAUGGUUUGUG
miR-15a-5p mimics antisense	CAAACCAUUAUGUGCUGCUAUU
mimics NC sense	UUCUCCGAACGUGUCACGUTT
mimics NC antisense	ACGUGACACGUUCGGAGAATT
GAPDH positive control sense	UGACCUCAACUACAUGGUUTT
GAPDH positive control antisense	AACCAUGUAGUUGAGGUCATT
**FISH probe sequences**	
miR-15a-5p-Cy3 probel	CACA+AACCATTA+TGTGCTGCTA
18S-Cy3 probel	CTGCCTTCCTTGGATGTGGTAGCCGTTTC
NC-Cy3 probel	TGCTTTGCACGGTAACGCCTGTTTT
has circ 0069382-FAM probel	G+TAAAGGAAACT+TTA+TAGAT+TAGAC
**Lentivirus insertion sequences**	
LV3-hsa-miR-15a-5p mimics	TAGCAGCACATAATGGTTTGTG
LV3-NC	TTCTCCGAACGTGTCACGT
**Antibodys**	
GAPDH	Cell Signaling Technology(2118S)
BTG2	proteintech (22339-1-AP)
BTG2	abcam (ab197362)
FAK	Cell Signaling Technology (3285T)
IgG (H+L) Secondary Antibody,HR	Invitrogen (32460)

<table>
<tr><th colspan="4">Table S6 Potential IRES for hsa_circ_0069382</th></tr>
<tr><th>Sequences producing significant alignments</th><th>Score (bits)</th><th>E Value</th><th>site</th></tr>
<tr><td>IRESite_Id:625 SINV1_IGRpred virus</td><td>29</td><td>3.5</td><td>Query: 89 aaaagaattttgaaga 104
| | | | | | | || || | | | | |
Sbjct: 7874 aaaagaattttgaaga 7859</td></tr>
<tr><td>IRESite_Id:342 Apaf-1 gene</td><td>28</td><td>12</td><td>Query: 250 ggattttcttgaggt 264
| | | || || | || | | | | |
Sbjct: 4225 ggattttcttgaggt 4211</td></tr>
<tr><td>IRESite_Id:1 HIF1a gene</td><td>26</td><td>39</td><td>Query: 325 tttgggtttaattc 338
||| | | | ||| | | || |
Sbjct: 2413 tttgggtttaattc 2400</td></tr>
<tr><td>IRESite_Id:637 EV71_1-748 virus</td><td>26</td><td>39</td><td>Query: 280 caacccaaattatg 293
| | | | | | | | ||| | | |
Sbjct: 3265 caacccaaattatg 3278</td></tr>
<tr><td>IRESite_Id:37 BVDV1_29-391 virus</td><td>26</td><td>39</td><td>Query: 468 tacgtattgggcaa 481
|| | | | | || | | | | | |
Sbjct: 30 tacgtattgggcaa 43</td></tr>
<tr><td>IRESite_Id:37 BVDV1_1-385 virus</td><td>26</td><td>39</td><td>Query: 468 tacgtattgggcaa 481
|| | | | || | | | | | | |
Sbjct: 30 tacgtattgggcaa 43</td></tr>
<tr><td>ESite_Id:9 plasmid pBi5_BVDV with functional BVDV1 IRES from B...</td><td>26</td><td>39</td><td>Query: 468 tacgtattgggcaa 481
|| | | | || | | | | | | |
Sbjct: 3981 tacgtattgggcaa 3994</td></tr>
<tr><td>IRESite_Id:18 plasmid ORF(delta386-901) with defective BVDV1_del...</td><td>26</td><td>39</td><td>Query: 468 tacgtattgggcaa 481
|| | | | || | | | | | | |
Sbjct: 3981 tacgtattgggcaa 3994</td></tr>
<tr><td>IRESite_Id:17 plasmid IIIe(delta323-337) with defective BVDV1_de...</td><td>26</td><td>39</td><td>Query: 468 tacgtattgggcaa 481
|| | | | || | | | | | | |
Sbjct: 3981 tacgtattgggcaa 3994</td></tr>
<tr><td>IRESite_Id:16 plasmid IIId(delta266-293) with defective BVDV1_de...</td><td>26</td><td>39</td><td>Query: 468 tacgtattgggcaa 481
|| | | | || | | | | | | |
Sbjct: 3981 tacgtattgggcaa 3994</td></tr>
<tr><td>IRESite_Id:15 plasmid IIIc(delta245-254) with defective BVDV1_de...</td><td>26</td><td>39</td><td>Query: 468 tacgtattgggcaa 481
|| | | | | || | | | | | |
Sbjct: 3981 tacgtattgggcaa 3994</td></tr>
<tr><td>IRESite_Id:22 plasmid IIIb-c(In237-244) with defective BVDV1_in2...</td><td>26</td><td>39</td><td>Query: 468 tacgtattgggcaa 481
|| | | | || | | | | | | |
Sbjct: 3981 tacgtattgggcaa 3994</td></tr>
</table>

<table>
<tr><td>IRESite_Id:21 plasmid IIIb(In225-228) with defective BVDV1_in225...</td><td>26</td><td>39</td><td>Query: 468 tacgtattgggcaa 481
|| | | | || | | | | | | | |
Sbjct: 3981 tacgtattgggcaa 3994</td></tr>
<tr><td>IRESite_Id:14 plasmid IIIb(delta208-223) with defective BVDV1_de...</td><td>26</td><td>39</td><td>Query: 468 tacgtattgggcaa 481
| | | || | || | | | | | | |
Sbjct: 3981 tacgtattgggcaa 3994</td></tr>
<tr><td>IRESite_Id:20 plasmid IIIa(In170-173) with defective BVDV1_in170...</td><td>26</td><td>39</td><td>Query: 468 tacgtattgggcaa 481
|| | | | || | | | | | | |
Sbjct: 3981 tacgtattgggcaa 3994</td></tr>
<tr><td>IRESite_Id:19 plasmid IIa-b(In102-105) with defective BVDV1_in10...</td><td>26</td><td>39</td><td>Query: 468 tacgtattgggcaa 481
|| | | | || | | | | | | | |
Sbjct: 3981 tacgtattgggcaa 3994</td></tr>
<tr><td>IRESite_Id:12 plasmid II(delta78-137) with defective BVDV1_delta...</td><td>26</td><td>39</td><td>Query: 468 tacgtattgggcaa 481
|| | | | | || | | | | | | |
Sbjct: 3981 tacgtattgggcaa 3994</td></tr>
<tr><td>IRESite_Id:23 plasmid Ib(In63-66) with functional BVDV1_in63-66 ...</td><td>26</td><td>39</td><td>Query: 468 tacgtattgggcaa 481
|| | | | | || | | | | | | |
Sbjct: 3981 tacgtattgggcaa 3994</td></tr>
<tr><td>RESite_Id:24 plasmid AUG(In375-378) with defective BVDV1_in375-...</td><td>26</td><td>39</td><td>Query: 468 tacgtattgggcaa 481
|| | | | | || | | | | | |
Sbjct: 3981 tacgtattgggcaa 3994</td></tr>
</table>

Table S7 Potential ORFs for hsa_circ_0069382					
**Label**	**Strand**	**Frame**	**Start**	**Stop**	**Length (nt | aa)**
ORF1	+	2	62	>673	612 | 203
ORF2	-	1	643	488	156 | 51
